# Antihypertensive medication classes and risk of incident dementia in primary care patients: a longitudinal cohort study in the Netherlands

**DOI:** 10.1016/j.lanepe.2024.100927

**Published:** 2024-05-15

**Authors:** Jakob L. Schroevers, Marieke P. Hoevenaar-Blom, Wim B. Busschers, Monika Hollander, Willem A. Van Gool, Edo Richard, Jan Willem Van Dalen, Eric P. Moll van Charante

**Affiliations:** a Department of General Practice, Amsterdam UMC, University of Amsterdam, Meibergdreef 9, 1105 AZ, Amsterdam, the Netherlands; b Department of Public & Occupational Health, Amsterdam UMC, University of Amsterdam, Meibergdreef 9, 1105 AZ, Amsterdam, the Netherlands; c Julius Center for Health Sciences and Primary Care, University Medical Center Utrecht, Universiteitsweg 100, 3584 CG, Utrecht, the Netherlands; d Department of Neurology, Donders Institute for Brain, Cognition, and Behaviour, Radboud University Medical Center, Geert Grooteplein Zuid 10, 6525 GA, Nijmegen, the Netherlands; e Department of Neurology, Amsterdam UMC, University of Amsterdam, Meibergdreef 9, 1105 AZ, Amsterdam, the Netherlands

**Keywords:** Dementia, Alzheimer's disease, Hypertension, Antihypertensive medication, Angiotensin hypothesis, Primary care

## Abstract

**Background:**

Hypertension is a modifiable risk factor for dementia affecting over 70% of individuals older than 60. Lowering dementia risk through preferential treatment with antihypertensive medication (AHM) classes that are otherwise equivalent in indication could offer a cost-effective, safe, and accessible approach to reducing dementia incidence globally. Certain AHM-classes have been associated with lower dementia risk, potentially attributable to angiotensin-II-receptor (Ang-II) stimulating properties. Previous study results have been inconclusive, possibly due to heterogeneous methodology and limited power. We aimed to comprehensively investigate associations between AHM (sub-)classes and dementia risk using large-scale continuous, real-world prescription and outcome data from primary care.

**Methods:**

We used data from three Dutch General Practice Registration Networks. Primary endpoints were clinical diagnosis of incident all-cause dementia and mortality. Using Cox regression analysis with time-dependent covariates, we compared the use of angiotensin-converting enzyme inhibitors (ACEi) to angiotensin receptor blockers (ARBs), beta blockers, calcium channel blockers (CCBs), and diuretics; and Ang-II-stimulating- to Ang-II-inhibiting AHM.

**Findings:**

Of 133,355 AHM-using participants, 5877 (4.4%) developed dementia, and 14,079 (10.6%) died during a median follow-up of 7.6 [interquartile range = 4.1–11.0] years. Compared to ACEi, ARBs [HR = 0.86 (95% CI = 0.80–0.92)], beta blockers [HR = 0.81 (95% CI = 0.75–0.87)], CCBs [HR = 0.77 (95% CI = 0.71–0.84)], and diuretics [HR = 0.65 (95% CI = 0.61–0.70)] were associated with significantly lower dementia risks. Regarding competing risk of death, beta blockers [HR = 1.21 (95% CI = 1.15–1.27)] and diuretics [HR = 1.69 (95% CI = 1.60–1.78)] were associated with higher, CCBs with similar, and ARBs with lower [HR = 0.83 (95% CI = 0.80–0.87)] mortality risk. Dementia [HR = 0.88 (95% CI = 0.82–0.95)] and mortality risk [HR = 0.86 (95% CI = 0.82–0.91)] were lower for Ang-II-stimulating versus Ang-II-inhibiting AHM. There were no interactions with sex, diabetes, cardiovascular disease, and number of AHM used.

**Interpretation:**

Among patients receiving AHM, ARBs, CCBs, and Ang-II-stimulating AHM were associated with lower dementia risk, without excess mortality explaining these results. Extensive subgroup and sensitivity analyses suggested that confounding by indication did not importantly influence our findings. Dementia risk may be influenced by AHM-classes’ angiotensin-II-receptor stimulating properties. An RCT comparing BP treatment with different AHM classes with dementia as outcome is warranted.

**Funding:**

10.13039/100009647Netherlands Organisation for Health, Research and Development (ZonMw); 10.13039/100016064Stoffels-Hornstra Foundation.

**Research in context**

**Evidence before this study**

Hypertension is a modifiable risk factor for dementia. Some antihypertensive medication classes might reduce dementia risk more than other classes, potentially through angiotensin-II-receptor stimulating properties. We searched PubMed for articles published in any language between 1977 and September 2023, using the terms and variations for “antihypertensive medication”, “angiotensin-converting enzyme inhibitors”, “angiotensin II receptor blockers”, “beta blockers”, “calcium channel blockers”, “diuretics”, “hypertension”, “blood pressure”, “dementia”, and “Alzheimer's disease”. We found various observational cohort studies and meta-analyses; however, the results on whether specific antihypertensive medication classes were more effective in lowering dementia risk than others varied. These inconsistent findings may be attributed to several factors, including choice of comparator (i.e. comparing to ‘non-users’ or ‘any other antihypertensive medication class users’, reducing overall contrast), use of single time-point (baseline) rather than dynamic drug exposures over time, not accounting for competing risk of death, and insufficient exploration of potential confounding by indication.

**Added value of this study**

In this study, addressing some of the potential methodological flaws from previous studies, we further strengthened evidence that among patients, all receiving some form of antihypertensive medication, angiotensin II receptor blockers, calcium channel blockers, and thiazide diuretics might lower dementia risk more than angiotensin-converting enzyme inhibitors, potentially due to angiotensin-II-receptor stimulating properties. This supports previous findings and aligns with the angiotensin hypothesis. These results cannot be explained by excess mortality. Lastly, we used time-dependent variables for both medication exposure and a wide range of confounders, reducing misclassification over time.

**Implications of all the available evidence**

Ideally, randomised controlled trials with head-to-head comparisons between antihypertensive medication classes are needed to corroborate these findings before further recommendations can be made. If replicated in an RCT, preferential prescription of one commonly used guideline-equivalent antihypertensive class over another may provide a cheap, safe, and accessible way to reduce dementia incidence in aging populations worldwide.

## Introduction

Approximately 55 million people worldwide have dementia. With global aging, this number is expected to increase to 153 million by 2050, making dementia prevention an international major health priority.^1^^,^^2^ Hypertension is an important risk factor for all-cause dementia, including Alzheimer's disease (AD),^2^^,^^3^ which is the cause of dementia in >70% of adults over the age 60.^4^ RCT and observational evidence suggests that blood pressure (BP) lowering using antihypertensive medication (AHM) reduces dementia risk in hypertensive individuals.^5^^,^^6^ Moreover, a recent network meta-analysis suggested that specific AHM-classes, particularly angiotensin receptor blockers (ARBs) and calcium channel blockers (CCBs), reduce dementia risk beyond their BP lowering effects.^7^ These AHM-classes are commonly used and considered equivalent to other AHM-classes for lowering BP by guidelines worldwide.^8^, ^9^, ^10^, ^11^ Thus, preferential prescription of these classes for lowering BP may provide an easily accessible and cost-effective method to reduce dementia risk globally. However, two recent, relatively small individual participant data (IPD) collaborations did not find significant differences in dementia risk between AHM-classes.^12^^,^^13^

Several factors may underlie this heterogeneity. Studies used different comparator categories, including non-users and “any other AHM-class”, instead of comparing individual AHM-classes directly.^7^^,^^12^^,^^13^ This may have diminished contrast and caused insufficient power to detect the approximated 10–30% risk differences between AHM classes.^7^ Furthermore, studies based on AHM exposure at single time points may not reflect the clinical reality, as AHM regimens are likely to change over time. The resulting potential exposure misclassification may attenuate class-specific associations, especially with extended follow-up durations,^14^ which are required when researching dementia, due to its gradual onset.^15^ Finally, studies often do not separately investigate subclasses of diuretics (thiazides/K-sparing/loop) and CCBs (dihydropyridine/non-dihydropyridine), despite these subclasses having considerably varying clinical indications and mechanisms of action.^8^, ^9^, ^10^^,^^16^ Recent studies suggest that specifically angiotensin-II-receptors type 2 and 4 (Ang-II) stimulating AHM-subclasses (ARBs, dihydropyridine CCBs, thiazides), lower dementia risk compared to Ang-II-inhibiting subclasses (angiotensin-converting enzyme inhibitors [ACEi], non-dihydropyridine CCBs, beta blockers).^14^^,^^17^, ^18^, ^19^ Not distinguishing between these subclasses in exposure or comparator groups may have further diminished contrast and increased heterogeneity based on differences in AHM-subclass usage between studies.

This study aims to address these issues by comprehensively investigating the associations between AHM-(sub)classes and incident dementia risk, using large-scale real-world primary care data, incorporating time-dependent diagnoses and medication changes from over 130,000 Dutch community dwelling, AHM-using older adults from 1988 to 2022. In addition, we explore the extent to which competing risk of mortality, and confounding by indication may influence these results.

## Methods

### Population

We used routine care data from three Dutch General Practice Registration Networks (GPRN) registered from January 1988 to December 2022. GPRNs record individuals’ demographics, medical history, and prescriptions, retrieved from electronic health records (EHR) of general practitioners (GP), >98% of the Dutch population is registered at a GPs-office. Diagnoses and medication use in GPRNs are considered representative of the Dutch population.^20^ We included all participants with any AHM use during the observed period who were aged ≥65 years when reaching an endpoint (dementia diagnosis, death, or deregistration), without further in-/exclusion criteria. Data use approval was obtained from each GPRN. Data were anonymously aggregated, requiring no ethical approval.

### Endpoints

The primary outcome was all-cause dementia using International Classification of Primary Care (ICPC) codes (Supplementary Methods 1). Dutch GPRNs include diagnoses made by hospital specialists (e.g. neurologists, geriatricians) following clinical guidelines. Hypertension is primarily managed by GPs. For a brief overview of the role of the GP in Dutch Healthcare, please refer to Supplementary Methods 2. All-cause mortality was collected as secondary outcome.^21^ Participants leaving the participating GPRNs (e.g., moving from the region) were censored at the deregistration date.

### AHM exposure

**Fig. 1:**
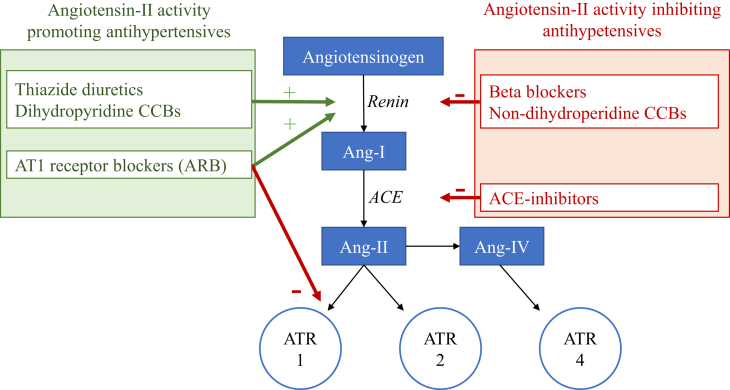
**Angiotensin hypothesis**. Thiazides and dihydropyridine calcium channel blockers (dihydropyridine CCBs) increase renin. Beta–blockers reduce β_1_-mediated renin production. Long-acting forms of verapamil nor diltiazem CCBs (non-dihydropyridine CCBs) affect or reduce renin. Renin generates angiotensin-I which is converted by angiotensin-converting enzyme (ACE) to angiotensin-II, which has physiological effects by binding to ATR1 or ATR2 or it may be metabolised to Ang-IV, which binds to ATR4. Angiotensin-converting enzyme inhibitors (ACE-Is) and angiotensin receptor-1 blockers (ARBs) impact on the Renine-Angiotensine System (RAS) via different Ang-II effects. ACE is reported to degrade Amyloid-β, a major component of dementia plaques in the brain. This degradation may be reduced by ACE-Is. ARBs selectively inhibit Ang-II at angiotensin-receptor 1 (ATR1) without inhibiting ACE. Ang-II and Ang-IV activity may protect from ischemia via activity at ATR2 and possibly ATR4. Ang-II and Ang-IV may directly affect memory. Green arrows and text: Ang-II stimulating antihypertensives; red: Ang-II inhibiting antihypertensives; blue boxes: Angiotensin peptides; blue circles: angiotensin receptors. Recreated from reference 17.

AHM-use was based on successive prescriptions using ATC codes. We investigated five main AHM-classes: ACEi, ARBs, beta blockers (BBs), CCBs and diuretics. All included ATC codes divided by class are described in Supplementary Methods 4. We distinguished between dihydropyridine and non-dihydropyridine CCBs, and thiazide(-like), loop- and potassium-sparing diuretics. Additionally, we compared Ang-II-stimulating AHM (ARBs, dihydropyridine CCBs and thiazide diuretics) to Ang-II-inhibiting AHM (ACEi, BB, non-dihydropyridine CCBs), adjusting for K-sparing and loop-diuretics, as these are not categorised as either Ang-II-stimulating or inhibiting (Fig. 1).^14^^,^^17^, ^18^, ^19^ Over 8 million prescriptions in 133,355 individuals were used to create a detailed, continuous medication overview for each patient. Within chronic medication regimens, gaps may occur between recurring prescriptions, since arrangements with pharmacies allow for multiple retrievals based on one prescription. Therefore, for chronic users with ≥3 successive prescriptions of an AHM-class, we assumed continuous exposure to that AHM-class from the first to the last prescription date.

### Covariates

Age, sex, diabetes, and history of myocardial infarction (MI) and stroke (haemorrhagic and ischaemic) were documented as potential confounders and modifiers. Post-hoc, congestive heart failure (CHF) was added to this list. All covariates were coded as “present” from time of first occurrence within the GP's EPD onwards.

### Statistical analysis

Associations between AHM-classes and incident dementia were calculated using Cox regression with time-dependent covariates.^22^^,^^23^ To account for possible dependencies between datasets, we included random terms for them. Additionally, we explored the inclusion of fixed terms for datasets, and random terms for the individual general practices within datasets to assess the robustness our results. Time since first registered AHM prescription was used as timescale, with incident dementia as outcome. Changes to AHM-classes were handled dynamically. If a person would change within a class (e.g. Lisinopril to Perindopril, both ACEi), exposure status would remain unchanged. If a person was prescribed a new class, either by adding a drug from another class to the existing class, or replacing it, exposure would change from the exact time-point the new class was added (e.g. adding Amlodipine to Perindopril after three years would change exposure from ACEi to ACEi and CCB from that moment onwards). All confounders were handled in a similar fashion. For example, if someone was diagnosed with diabetes after three years of follow-up, exposure to diabetes would start at time of diagnosis, leaving the person unexposed to diabetes for the first three years. Only active AHM-users were included. To facilitate comparison between the five AHM-classes, we decided to use one class as reference category. The choice fell on ACEi because, compared to the four other AHM-classes, they showed the highest dementia incidence, and were consistently associated with the highest dementia risk in our Cox regression models, consistent with previous studies.^7^^,^^13^ Use of ARBs, BBs, CCBs, and diuretics were the main predictors, adjusted for the total number of AHM-classes used including ACEi, thereby resulting in hazard ratios (HR) for use of these AHM types compared to ACEi-use (Supplementary Methods 3). Model 1 included baseline age, and sex as covariates (detailed effects in Supplementary Methods 5). Model 2 additionally included diabetes, MI, and stroke. Model 3 corrected for all available covariates, including CHF. Proportional hazards assumptions were assessed using visual inspection of Schoenfeld residuals.

In Cox models, the exposure at the time of event determines the HR. However, prescription stop-dates in the GPRN may not always precisely reflect end-of-use. This may systematically differ per AHM-class, and indication. To recompense, we set the end date of all AHM-classes prescribed during the last recorded year to the end of the last prescription in that year. Second, individuals with incipient dementia may withdraw from care or stop medication, resulting in longstanding AHM-users not having prescriptions at the actual dementia diagnosis date. To address this, the last recorded AHM-regimen was extended up to two years until censoring (Supplementary Figure S1). We assessed how these two adjustments influenced results in two sensitivity analyses, leaving out each modification respectively. Thirdly, we performed a sensitivity analysis in which AHM-class exposure was determined relative to the total AHM exposure time, because AHM may exert their effects on dementia risk over a prolonged period, prior to the date of diagnosis. To account for reverse causation, we tested for AHM exposure cut-off at ≥25%, ≥50%, and ≥75% of cumulative exposure time. We conducted two additional post-hoc analyses. One excluding individuals who developed dementia within one year of the last AHM regimen change, and another excluding those who developed dementia within one year of starting AHM treatment in our dataset. Lastly, to approach the association with dementia with and without a vascular component, we repeated the main analysis in participants with major cardiovascular disease (CVD), including myocardial infarction (MI) and stroke, and in those without this CVD comorbidity.

Subgroup analyses were performed, stratifying analyses according to 1.) sex, as studies have suggested that the influence of the renin-angiotensin system differs between men and women^24^; 2.) baseline age (<70 years, 70–80 and > 80 years) as the major predictor of dementia risk; 3.) history of CVD (MI and/or stroke), and 4.) diabetes, as both these disease histories influence dementia risk as well as the preferential AHM-class for treatment. Furthermore, we stratified analyses according to the total number of AHM-classes used simultaneously, since this may be related to hypertension severity and comorbidity (and thereby dementia risk), and some AHM-classes may more often be prescribed at later stages and/or in combination. Finally, during the early years of this cohort (1988–2012), Dutch primary care guidelines recommended both ARBs and CCBs as later steps in hypertension treatment, whereas ACEi and diuretics were first line of choice. Due to this recommendation, ARBs and CCBs may have been reserved for patients with more severe hypertension during these early years of observation. In the latter years of this study (2012–2022), according to the updated guideline on CVD prevention in clinical practice, all AHM-classes were regarded as equivalent therapeutic options for treatment of uncomplicated hypertension.^25^ To assess the consequence of this guideline-change, both time periods were studied separately, using 2015 as cut-off to ensure that the 2012 guideline change was adopted by all GPs.

We assessed the competing risk of death using a cause-specific hazard approach, repeating the main analysis for the outcomes of mortality and dementia/mortality combined. We chose this approach over a sub-distribution hazard (e.g. Fine–Gray analyses) for several reasons: 1) our research question is an etiological one, for which the cause-specific hazard approach is most appropriate,^26^^,^^27^ 2) we were not aiming to create a prediction model, able to predict the expected number of dementia cases for different AHM groups over a projected period, for which the sub distribution hazard approach is appropriate,^26^ and 3.) analysis of time-varying covariates in Fine–Gray models is problematic because it requires making assumptions about the exposure post-censoring for the competing event.^28^ Nevertheless, we did explore the potential impact of the sub-distribution hazard approach on our results. Additionally, to explore how subgroups might influence our mortality findings, we repeated all subgroup analyses with mortality as endpoint.

Data were analysed using R 4.1.2 package ‘survival’.^22^^,^^29^

### Role of the funding source

The funding source had no role in study design, data collection, data analysis, interpretation, writing, or submitting this article.

## Results

**Fig. 2:**
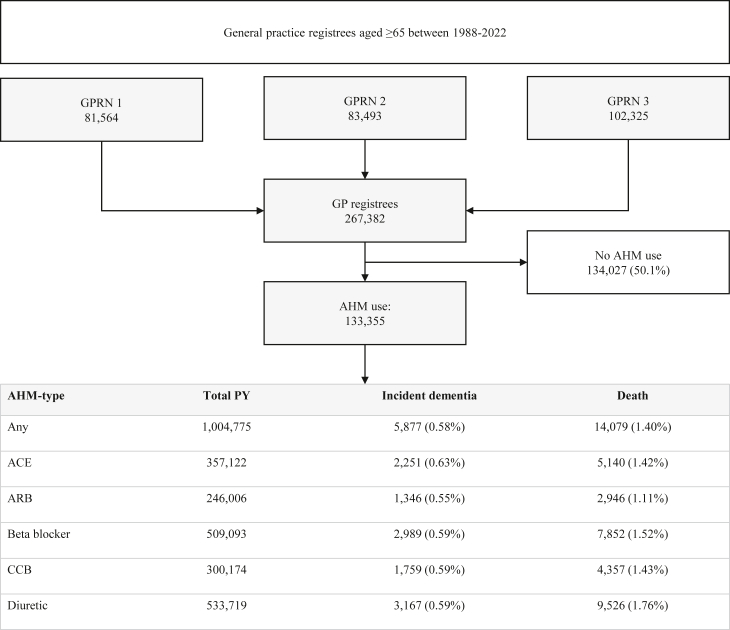
**Flowchart data-extraction from the three general practice registration networks (GPRN).** Only patients aged 65 or above at any point between 1988 and 2022 were extracted. Abbreviations: GP, general practice; AHM, antihypertensive medication; ACE, angiotensin converting enzyme inhibitor; ARB, angiotensin receptor blocker; BB, beta blocker; CCB, calcium channel blocker; Diu, diuretic.

Out of 267,382 registrants aged ≥65 between 1988 and 2022, 133,355 (49.9%) used AHM and were included (Fig. 2). Median age of entry into the cohort was 68 years (Table 1), 54.7% were women, and median follow-up was 7.6 years (IQR = 4.1–11.0), yielding 1,004,775 person years (PY). During the observed period, 5877 (4.4%) participants developed dementia, and 14,079 (10.6%) died. Diabetes occurred in 36,613 (27.5%), MI in 19,139 (14.4%) stroke in 12,096 (9.1%), and congestive heart failure in 14,798 (11.1%) participants. Population characteristics were similar between GPRNs (Supplementary Table S1). Common AHM-class combinations at baseline and during the last year of observation are depicted in Supplementary Table S2.

**Table 1:** Population characteristics at baseline, and after follow-up.

Baseline	Total population	ACEi	ARB	Beta blocker	CCB	Diuretic	Ang-II-stimulating AHM	Ang-II-inhibiting AHM
	N = 133,355	N = 34,545	N = 17,669	N = 50,285	N = 23,860	N = 51,076	N = 63,026	N = 80,305
Women, n (%)	72,884 (54.7)	15,770 (45.7)	9665 (54.7)	27,271 (54.2)	12,324 (51.7)	30,74 (60.2)	35,944 (57.0)	41,531 (51.7)
Baseline age, median [IQR]	68.2 [62.0–75.8]	68.2 [62.3–75.4]	68.7 [62.6–76.1]	67.9 [61.5–75.6]	69.2 [63.4–76.5]	69.7 [63.0–77.8]	68.3 [62.4–75.6]	67.9 [61.7–75.4]
<65 years, n (%)	49,279 (61.4)	12,491 (36.2)	6177 (35.0)	19,333 (38.4)	7632 (32.5)	16,607 (32.5)	22,763 (36.1)	30,592 (38.1)
65–75 years, n (%)	47,765 (59.5)	13,038 (37.7)	6491 (36.7)	17,557 (34.9)	9178 (38.5)	17,806 (34.9)	23,440 (37.2)	28,807 (35.9)
75–85 years, n (%)	27,465 (34.2)	6944 (20.1)	3868 (21.9)	10,170 (20.2)	5418 (22.7)	11,856 (23.2)	13,064 (20.7)	16,044 (29.0)
≥85 years, n (%)	8846 (11.0)	2072 (6.0)	1133 (6.4)	3225 (6.4)	1632 (6.8)	4807 (9.4)	3759 (6.0)	4862 (6.1)
History of type 2 diabetes, n (%)	26,655 (20.0)	10,207 (29.5)	4611 (27.0)	8809 (17.5)	5218 (21.9)	10,831 (21.2)	13,643 (21.6)	17,194 (21.4)
History of myocardial infarction, n (%)	13,793 (10.3)	4989 (14.4)	1817 (10.3)	8110 (16.1)	2658 (11.1)	3767 (7.4)	4544 (7.2)	10,942 (13.6)
History of stroke, n (%)	9075 (6.8)	2169 (6.3)	1142 (6.5)	3624 (7.2)	1761 (7.4)	3282 (6.4)	4183 (6.6)	5551 (6.9)
History of congestive heart failure, n (%)	5846 (4.4)	1854 (5.4)	803 (4.5)	2641 (5.3)	917 (3.8)	3681 (7.2)	1784 (2.8)	3756 (4.7)
**At time of censoring**	**N** = **133,355**, **PY** = **1,004,775**	**N** = **46,362**, **PY** = **357,122**	**N** = **30,466**, **PY** = **246,006**	**N** = **63,213**, **PY** = **509,093**	**N** = **42,339**, **PY** = **300,174**	**N** = **67,611**, **PY** = **533,719**	**N** = **78,356**, **PY** = **631,650**	**N** = **93,020**, **PY** = **727,829**
Censoring age, median [IQR]	76.5 [70.7–83.6]	76.1 [70.6–83.0]	76.2 [70.8–83.0]	76.9 [71.1–84.0]	76.0 [70.6–82.9]	77.9 (71.6–85.1]	76.5 [70.7–83.6]	76.4 [70.8–83.5]
<65 years, n (%)	497 (0.4)	152 (0.3)	120 (0.4)	204 (0.3)	140 (0.3)	240 (0.4)	305 (0.4)	323 (0.3)
65–75 years, n (%)	58,190 (43.6)	17,162 (37.0)	13,421 (44.1)	26,455 (41.9)	19,100 (45.1)	26,061 (38.5)	35,307 (45.1)	40,537 (43.6)
75–85 years, n (%)	46,601 (34.9)	16,500 (35.6)	11,279 (37.0)	22,845 (36.1)	15,202 (35.9)	24,144 (35.7)	28,007 (35.7)	33,025 (35.5)
≥85 years, n (%)	28,049 (21.0)	8852 (19.1)	5646 (18.5)	13,709 (21.7)	7897 (18.7)	17,166 (25.4)	14,737 (18.8)	19,135 (20.6)
Type 2 diabetes, n (%)	36,613 (27.5)	16,151 (34.8)	10,034 (32.9)	18,134 (28.7)	13,275 (31.4)	20,833 (30.8)	23,712 (30.3)	27,476 (29.5)
Myocardial infarction, n (%)	19,139 (14.4)	8428 (18.2)	4664 (15.3)	13,400 (21.2)	6066 (14.3)	9225 (13.6)	9600 (12.3)	16,435 (17.7)
Stroke, n (%)	12,096 (9.1)	3945 (8.5)	2817 (9.2)	5966 (9.4)	4155 (9.8)	5806 (8.6)	7119 (9.1)	8534 (9.2)
Congestive heart failure, n (%)	14,798 (11.1)	5876 (12.7)	3627 (11.9)	9551 (15.1)	3801 (9.0)	12,380 (18.3)	6667 (8.5)	11,739 (12.6)
Incident dementia, n (%)	5877 (4.4)	2251 (4.9)	1346 (4.4)	2989 (4.7)	1759 (4.2)	3167 (4.7)	3491 (4.5)	4312 (4.6)
Incident mortality, n (%)	14,079 (10.6)	5140 (11.1)	2946 (9.7)	7852 (12.4)	4357 (10.3)	9526 (14.1)	7330 (9.4)	10,409 (11.2)

Baseline is the moment a participant enters a GPRN. Due to combination therapy, a participant may represented in multiple AHM-groups. Ang-II-stimulating/inhibiting AHM were analysed separately from individual AHM-classes. AHM, antihypertensive medication; ACEi, angiotensin-converting enzyme inhibitor; ARB, angiotensin receptor blocker; CCB, calcium channel blocker; Ang-II, angiotensin-II, angiotensin II type 2 receptor.

### Main outcomes

During a total of 357,122 PY of ACEi-use, 2251 individuals developed dementia (Table 2), yielding an incidence rate of 6.3‰. This was 5.5‰ for ARB-use (1346/246,006) and 5.9‰ for BBs (2989/509,093), CCBs (1759/300,174), and Diuretics (3167/533,719).

**Table 2:** Associations of AHM-use with dementia and mortality.

AHM-class	Dementia			Mortality			Dementia/Mortality		
	Cases/PY n(‰)	HR (95% CI)	P-value	Cases/PY n (‰)	HR (95% CI)	P-value	Cases/PY n (‰)	HR (95% CI)	P-value
ACEi	2251/357,122 (6.3)	*Reference*		5140/361,994 (14.2)	*Reference*		7662/357,122 (21.5)	*Reference*	
ARB	1346/246,006 (5.5)	0.86 (0.80–0.92)	<0.001	2946/248,697 (11.9)	0.83 (0.80–0.87)	<0.001	4443/246,006 (18.1)	0.85 (0.81–0.88)	<0.001
Beta blocker	2989/509,093 (5.9)	0.81 (0.75–0.87)	<0.001	7852/515,410 (15.2)	1.21 (1.15–1.27)	<0.001	11,225/509,093 (22.1)	1.06 (1.02–1.11)	<0.001
CCB	1759/300,174 (5.9)	0.77 (0.71–0.84)	<0.001	4357/304,486 (14.3)	1.04 (0.99–1.10)	0.15	6272/300,174 (20.9)	0.96 (0.92–1.00)	0.04
Diuretic	3167/533,719 (5.9)	0.65 (0.61–0.70)	<0.001	9526/541,233 (17.6)	1.69 (1.60–1.78)	<0.001	13,157/533,719 (24.7)	1.26 (1.21–1.31)	<0.001
Ang-II-inhibiting	4312/727,829 (5.9)	*Reference*		10,409/738,051 (14.1)	*Reference*		14,290/727,691 (19.6)	*Reference*	
Ang-II-stimulating	3491/631,650 (5.5)	0.88 (0.82–0.95)	<0.001	7330/639,836 (11.5)	0.86 (0.82–0.91)	<0.001	10,511/631,568 (16.6)	0.87 (0.84–0.91)	<0.001

Hazard ratio's (HR) for incident outcomes according Cox regression with time varying covariates. HRs present model 2, adjusting for age and sex at baseline, and number of AHM-classes simultaneously used, type 2 diabetes, myocardial infarction, and stroke as time dependent variables. Analyses for Ang-II inhibiting versus stimulating AHM were additionally adjusted for K-sparing & Loop diuretics as both subclasses are not represented in either Ang-II-stimulating or Ang-II-inhibiting AHM. Ang-II-inhibiting AHM include: ACEi, Beta blocker & non-dihydropyridine CCB; AngII-stimulating AHM: ARB, dihydropyridine CCB & Thiazide(like) diuretics. We found no evidence for non-proportionality in our Cox models according to the distribution of the Schoenfeld residuals. Cases/PY represent the total number of incident cases (Cases) that occurred during the total person years (PY) of exposure observed for each class of interest. Individual participants may be represented in multiple AHM-classes in case of combination therapy and medication switching over time. Abbreviations: AHM, antihypertensive medication; HR, hazard ratio; CI, confidence interval; ACEi, angiotensin-converting enzyme inhibitor; ARB, angiotensin receptor blocker, CCB, calcium channel blocker; Ang-II, angiotensin II type 2 receptor.

Described below are Cox regression analyses results for the fully adjusted Model 2. Compared to ACEi (Table 2), dementia risk was lower for ARBs (HR = 0.86; 95% CI = 0.80–0.92), BBs (HR = 0.80; 95% CI = 0.73–0.87), CCBs (HR = 0.77; 95% CI = 0.71–0.84), and diuretics (HR = 0.65; 95% CI = 0.61–0.70). Furthermore, dementia risk was lower for Ang-II-stimulating (HR = 0.88; 95% CI = 0.82–0.95) versus Ang-II-inhibiting AHM. Dementia risk was similar within CCB subclasses (dihydropyridine/non-dihydropyridine), and within diuretic subclasses (thiazide/loop/K-sparing) versus ACEi (Supplementary Table S3).

Regarding the competing risk of death, BBs (HR = 1.21; 95% CI = 1.15–1.27) and diuretics (HR = 1.69; 95% CI = 1.60–1.78) were associated with significantly higher-, CCBs with similar- (HR = 1.04; 95% CI = 0.99–1.10), and ARBs with significantly lower (HR = 0.83; 95% CI = 0.80–0.87) mortality risk versus ACEi. For the combined outcome of ‘dementia/mortality’, only ARBs (HR = 0.85; 95% CI = 0.81–0.88), and CCBs (HR = 0.96; 95% CI = 0.92–1.00), had a lower dementia/mortality risk than ACEi. For Ang-II-stimulating AHM, mortality risk (HR = 0.86 95% CI = 0.82–0.91) and dementia/mortality risk (HR = 0.87; 95% CI = 0.84–0.91) was lower compared to Ang-II-inhibiting AHM (Table 2). Within diuretic subclasses, mortality risk was lower for thiazides (HR = 0.87; 95% CI = 0.83–0.93), and higher for loop (HR = 3.05; 95% CI = 2.89–3.22) and K-sparing (HR = 1.50; 95% CI = 1.42–1.59) diuretics versus ACEi (Supplementary Table S3). Additionally adjusting for CHF did not change results for dementia, and yielded slightly lower point estimates for mortality for diuretics compared to model 2 (Supplementary Table S4).

### Sensitivity and subgroup analyses

Extending AHM final year prescriptions (Supplementary Table S5), and censoring at last prescription's end date (Supplementary Table S6) gave similar results. Different methods of adjusting for clustering did not change results (Supplementary Table S7). Defining AHM exposure as ≥25%, ≥50%, and ≥75% of cumulative exposure time slightly attenuated HRs (Supplementary Table S8). Results were similar when excluding individuals who developed dementia within one year of either the last change in AHM regimen, or initiation of AHM treatment (Supplementary Table S9). Associations between AHM-classes and dementia were stronger in participants with-compared to participants without major CVD, except for BB (Supplementary Table S10).

Results for dementia were similar for subgroups stratified by sex, diabetes, CVD, and total number of AHM-classes used simultaneously (Supplementary Tables S11 and S12), except for slightly lower dementia HRs for BBs with CVD (HR = 0.72; 95% CI = 0.64–0.81) versus without CVD (HR = 0.80; 95% CI = 0.73–0.87) (p-interaction = 0.03). In individuals with CHF, dementia risk for ARBs (HR = 1.02; 95% CI = 0.87–1.20) and BBs (HR = 1.02; 95% CI = 0.87–1.19) was comparable with ACEi. Stratified by baseline age (Supplementary Table S13), dementia HRs for ARBs and BBs versus ACEi attenuated with higher age (p-trend ≤0.02). Results were similar for individuals initiating AHM treatment before versus after 2015 (three years post guideline change), except for dementia HRs for BBs being neutral if prescribed after 2015 (Supplementary Table S14). Supplementary Tables S15–S18 present results for subgroup analyses for mortality, and for competing risk according to sub-distribution hazards (i.e., Fine–Gray).

## Discussion

In this observational cohort study, using continuous real-world AHM-exposure data from 133,355 AHM-using primary care patients with 5877 incident dementia cases during one million person-years, use of ARBs (HR = 0.86), BBs (HR = 0.81), CCBs (HR = 0.77) and diuretics (HR = 0.65), was associated with 14–35% lower dementia risk, compared to ACEi. These associations did not coincide with significantly increased mortality risks for ARBs (HR = 0.83) and CCBs (HR = 1.04), meaning that the lower dementia rates for these AHM-classes cannot be attributed to excess mortality. For BBs, excess mortality (HR = 1.21) counterbalanced lower dementia risk. For diuretics, excess mortality (HR = 1.69) exceeded lower dementia risk. This is substantiated when looking at the composite outcome dementia/mortality, where only ARBs (HR 0.85) and CCBs (HR 0.96) perform better than ACEi. Fine–Gray analysis yielded largely similar results to those of our main analysis. Dementia risks were similar for thiazides, K-sparing, and loop diuretics. However, only for thiazides, lower dementia risk (HR = 0.75) coincided with lower mortality risk (HR = 0.87). Ang-II-stimulating AHM were associated with 12% lower dementia risk (HR = 0.88) compared to Ang-II-inhibiting AHM, without excess mortality (HR = 0.86, HR = 0.87 for dementia/mortality combined).

Results were stable when adjusting for additional covariates, across sensitivity analyses, and in subgroups of sex, diabetes, CVD, and number of AHM-classes used simultaneously. In individuals with CHF, dementia risk for ARBs and BBs was comparable to ACEi, but mortality risk remained lower for ARBs. The lower dementia risks associated with ARBs and beta-blockers compared to ACEi slightly attenuated with increasing age. This may be a chance finding. Alternative explanations are speculative. Possibly, some classes, such as ARBs, rely more on neuroprotective properties that are particularly exerted before extensive neuropathological changes associated with late life dementia develop, thereby potentially extending their therapeutic benefits, especially in younger age groups. Alternatively, ARBs might particularly reduce the risk of dementia with (micro-)vascular origin, which could represent a larger proportion of dementia cases in younger patients.^30^ This hypothesis is supported by our analyses focusing on dementia in participants with major CVD. For BB, their predominant role in CVD, accompanied by subsequent increased mortality estimates, complicates interpretation. These age-related disparities may merit further investigation in future longitudinal studies.

### Literature comparison

Similar to these results, a recent systematic review (n ˜ 649,000 AHM-users, 19,600 dementia cases, 27 studies) that used network meta-analysis to compare AHM classes directly, found 12–14% lower dementia risks for ARBs (RR = 0.88) and CCBs (RR = 0.86) versus ACEi.^7^ The difference for diuretics (RR = 0.95) versus ACEi was less clear.^7^ Two recent smaller IPD meta-analyses found no clear differences in dementia risk between AHM-classes but did not compare these directly to each other. One (n ≈ 7500 AHM-users, 650–750 dementia cases) found no significant differences for individual AHM-classes versus non-users or placebo.^12^ However, compared to the point estimate for ACEi (OR = 1.14), those for ARBs (OR = 0.95), CCBs (OR = 0.92), and diuretics (OR = 0.84) yielded15-25% lower risk of dementia, comparable to our findings, but not for BBs (OR = 1.17). The second IPD (n ≈ 7800 AHM-users, up to 1250 dementia cases) found no significant differences in dementia risk between individual AHM-classes versus all other AHM-classes combined, using propensity scores to adjust for potential indication bias.^13^ Nevertheless, the reported point estimates for ARBs (HR = 0.88) diuretics (HR = 0.95) and BBs (HR = 0.95) yielded 15–20% lower dementia risk than ACEi (HR = 1.11), consistent with our findings, although less so for CCBs (HR = 1.04: 7% lower). Point estimates remained largely the same for Alzheimer's dementia compared to those of all-cause dementia.^13^ Class-differences in these IPDs may have been significant had AHM-classes been compared directly to each other, rather than to any- or non-users. Two other recent meta-analyses that compared ACEi and ARBs with any other AHM-classes reported the lowest risk of dementia and AD for ARBs.^31^^,^^32^ In a similar primary care setting in Germany, an observational study among individuals with hypertension, which matched participants with and without dementia, observed that users of ARBs had a 6% lower risk of dementia compared to those using ACEi.^33^ None of these studies provided estimates of associated mortality for these AHM-classes, so the potential influence of excess mortality in these meta-analyses cannot be evaluated.

More recent studies specifically focused on Ang-II-stimulating versus Ang-II-inhibiting medication. In the first three,^17^, ^18^, ^19^ Ang-II-stimulating AHM-use was associated with a 24–40% lower risk of incident dementia^17^^,^^19^ or MCI/probable dementia^18^ compared to Ang-II-inhibiting AHM, without excess mortality. Most recently and comprehensively, a study in approximately 58,000 Medicare beneficiaries with newly discovered hypertension with 2000 incident dementia cases during 12 years of follow-up, found 16% lower AD and related dementias risk associated with Ang-II-stimulating versus Ang-II-inhibiting AHM-use.^19^ Notably, this study used Cox regression with time-dependent exposure, similar to our study. It did not report on mortality risk.

Altogether, AHM-classes appear to have differential associations with dementia risk. Lower risks for ARBs versus ACEi are among the most consistently reported, which is particularly noteworthy because these classes have identical indications, such as BP treatment in diabetes.

### Mechanisms

Although concurrent mortality data are relatively sparsely reported, associations for ARBs, CCBs and thiazide (like) diuretics with lower dementia risk do not seem attributable to excess mortality, while those for BBs and other diuretics do. This is understandable because BBs, loop- and K-sparing diuretics are often prescribed for life-limiting conditions including MI, CHF, and renal insufficiency.^34^ Conversely, the lower mortality risk observed among Ang-II-stimulating users could also be attributed to unknown patient characteristics, such as ethnicity and socioeconomic status, with potentially healthier participants more often prescribed specific classes (e.g. ARBs), leading to lower mortality rates in those groups.^35^^,^^36^

Several hypotheses suggest how individual AHM-classes might reduce dementia risk beyond BP lowering effects. For example, dihydropyridine CCBs may prevent neuronal cell death and AD neuropathology by regulating cellular calcium influx,^37^, ^38^, ^39^ and ARBs may reduce inflammation, oxidative stress, and AD neuropathology by improving cerebral blood flow.^39^^,^^40^ The more recent “angiotensin hypothesis” suggests that several AHM-classes lower dementia risk by stimulating the angiotensin-II-receptors type (ATR) 2 and 4, involved in cerebral ischemia and memory function (Fig. 1).^17^ ARBs directly block ATR1, increasing ATR2 and ATR4 stimulation, and upregulating angiotensin-II production. Thiazide diuretics and dihydropyridine CCBs stimulate ATR2 and ATR4 by increasing renin and thereby angiotensin-II production. BBs and non-dihydropyridine CCBs decrease renin and thereby angiotensin-II production. Finally, ACEis inhibit angiotensin-II production, inhibiting ATR2 and ATR4, and may also decrease cerebral Amyloid-Beta degradation wherein ACE is involved. This would fit with ACEi generally being associated with the highest dementia risk, ARBs versus ACEi most consistently with lower dementia risk, and the consistent results of studies evaluating Ang-II-stimulating versus inhibiting medication, discussed above. Our results do not fully support the angiotensin hypothesis. This could be attributed to residual confounding within the observational data or to other, as-yet-unknown mechanisms that might also influence the differential associations between antihypertensive medication classes and the risk of dementia.

### Strengths and limitations

This study has several strengths. First, we used a very large sample of community-dwelling older AHM-users, allowing for comprehensive subgroup and sensitivity analyses. Second, we studied exposures continuously rather than only at baseline yielding more accurate associations, especially with the protracted follow-up required for studying incident dementia. Third, we compared individuals who used different types of AHM directly to each other. Therefore, all included subjects had hypertension, and used antihypertensive drugs. Consequently, our analyses were not affected by differences in dementia risks attributable to hypertension status (i.e. non-hypertensive individuals likely have lower dementia risk), health seeking behaviour (i.e. higher dementia risks in untreated hypertensive individuals), or access to anti-hypertensive drugs. Fourth, because Dutch GPs actively gather and maintain their patients' health data in the GPRNs used in our study, we have relatively accurate real-world data with very low drop-out, minimizing the risk of observational biases. Fifth, because nearly all community-dwelling individuals in the Netherlands are registered with an GP, our sample is generally representative of the Dutch community-dwelling population,^20^ with the limitation that our participating GPRNs were located in the urbanised areas of Amsterdam, and Utrecht. Dutch GP's EHRs also contain diagnoses provided by other physicians, including hospital specialists, resulting in excellent specificity (6 studies, median 100%, range 78–100) for mild, and moderate to severe dementia. Sensitivity, however, is limited (up to 60%).^41^^,^^42^ Last, we extensively investigated individual AHM-classes, subclasses, and Ang-II categorizations, including concurrent associations for mortality. Thereby, this paper provides the most elaborate, comprehensive, and adequately powered evidence on differential associations for AHM-classes with dementia risk to date.

Unfortunately, we were unable to explore dementia subtypes due to data limitations. However, the likelihood of diagnostic imprecision varying with the class of AHM used is considered low, since the large majority of late-life dementia diagnoses concerns AD. This is supported by a large meta-analysis, which revealed similar hazards for both all-cause dementia and AD for different subclasses of AHM.^13^ From a clinical perspective, many patients diagnosed with AD also have some form of cerebrovascular comorbidity, hence could probably also be considered to harbour to a varying degree a vascular factor in addition to the neurodegenerative component. A further limitation is potential confounding by indication, which may have affected AHM prescription patterns in several ways. In the present Dutch GP guidelines, all antihypertensive medication (AHM) classes are considered equivalent treatments for uncomplicated hypertension. In cases of insufficiently controlled hypertension, it is recommended to add another AHM-class, instead of increasing dosages of the current regimen, to avoid adverse drug reactions (ADR). Under certain conditions, including diabetes, CVD, and CHF, specific AHMs are preferred. These preferences do not seem to have affected our main findings, as adjusting and stratifying for age, diabetes, CVD, and CHF hardly changed results. Inclusion of additional covariates, such as ethnicity and socioeconomic status could have further mitigated confounding by indication, although we believe the comorbidities incorporated in our analyses were the most relevant for the study objective. Our study relied on prescription data. Unknown patient characteristics, including treatment adherence and ADR may have influenced our results. Adherence can be adversely affected by ADR, which are common among users of AHMs. Older adults, in particular, are vulnerable to experiencing ADR due to polypharmacy. Consequently, they may experience decreased treatment adherence. For instance, ACEi and ARBs may cause kidney failure, especially when paired with diuretics or each other. BBs can induce fatigue and bradycardia, particularly when used alongside other negative chronotropic drugs, such as non-dihydropyridine CCBs. Non-dihydropyridine CCBs are associated with constipation, while dihydropyridines often lead to lower limb oedema, sometimes misinterpreted and mistakenly treated as heart failure. Diuretic-use may result in electrolyte imbalances, increased risk of diabetes when combined with BBs, or urinary incontinence. If more prevalent in specific classes, ADR may have resulted in increased inter-class switching and decreased adherence, which may have influenced results. Despite acknowledging the importance of considering ADR in older adults, most international hypertension guidelines, such as the American and Dutch guidelines, recommend the same five AHM classes for both younger and older individuals (aged >60), with British guidelines excluding BBs. This suggests that there is insufficient evidence for significant differences in ADR incidences among AHM-classes. Although current guidelines do not explicitly endorse a preference for specific classes with increasing hypertension severity or treatment resistance, GPs may prefer prescribing AHM classes in a specific order. Nevertheless, this appears to have had minimal impact, as the results were similar when stratified by the number of AHM used concurrently. Dihydropyridine CCBs, and non-selective BBs are preferred for hypertensive emergencies, however, these treatments are generally short lasting and administered in hospitalised settings, limiting their influence in primary care data. Dutch GP guidelines before 2012 recommended initiating primary hypertension treatment with thiazides or CCBs.^25^ Thus, these medications may have been particularly used in longstanding, relatively uncomplicated cases. However, associations were not attenuated for first-time users after 2015 except for BBs, suggesting that pre-existing AHM-class prescription preferences from before 2015 did not influence our results for ARBs, CCBs and thiazides. In the first half of our observational study, some classes, for instance ARBs, were still under patent resulting in higher prices, potentially limiting access to individuals with lower (social-) economic status. Nevertheless, results were similar when comparing prescription data before and after 2015. More importantly, in Dutch healthcare, all standard care including medication, is covered by mandatory health insurance, suggesting that overall impact of socioeconomic background on outcomes may have been limited. Reverse causation, for instance through switching antihypertensive regimens shortly before dementia diagnosis, may have occurred due to factors such as ease of use, adherence concerns, or ADR. However, it is unlikely that physicians systematically changed AHM regimens to a specific class before dementia diagnosis. Moreover, results for different proportions of AHM exposure were similar. Therefore, if reverse causation were a major factor, substantial differences would have been expected between the results of these exposure time analyses and those of the main analyses, as participants would have switched medications relatively shortly before dementia diagnosis. However, these exposure analyses are less informative regarding reverse causation if individuals with a greater dementia risk are also more susceptible to ADR, and are therefore more likely to use AHM-classes with fewer ADR or to be less adherent to these classes. Despite the assurances above, we cannot fully exclude residual confounding by, for example, unknown GP preferences or patient characteristics differentially affecting dementia risk. Another limitation may come from using regular care data. Although actively maintained by GPs, these may have relative inaccuracies compared to data from purpose-designed longitudinal studies with protocolised measurements by research personnel. Moreover, potential underestimation of dementia diagnoses may have played a role in our analyses, given the low level of sensitivity in GP-data (up to 60%), especially in mild dementia cases.^42^ It is unlikely that these inaccuracies systematically differed between AHM classes, biasing results towards neutral rather than exaggerated differences. Diagnoses made prior to entering a GPRN were available, however, medication were not. Therefore, some individuals that used and permanently stopped AHM prior to registering at a GPRN were not included in our dataset. Finally, the absence of blood pressure data in our model may be considered a potential limitation. However, significant proportions of missing data and potential bias in existing measurements towards higher risk would have hindered imputation of adequate time-dependent modelling necessary to account for the multitude of medication changes over time.^43^ Furthermore, BP lowering effects are considered more or less equal between AHM-classes, and studies adjusting for BP levels reported similar results.^9^^,^^10^^,^^25^^,^^44^ Moreover, a large meta-analysis reported that the association between blood pressure lowering with AHM and lower risk of dementia or cognitive decline was not affected by baseline blood pressure or cumulative systolic blood pressure change.^5^ Finally, in a different cohort with similar characteristics where BP values were available, we found no apparent differences in BP values between AHM-classes.^14^ Therefore, we expect that the differential associations in our study represent class-specific mechanisms affecting dementia risk beyond BP lowering effects.

### Conclusion

ARBs, CCBs, and thiazide diuretics were associated with lower dementia incidence rates compared to ACEi-use, without excess mortality. Combined with previous studies, our study makes a compelling case for differential associations between AHM-classes and dementia risk, particularly for lower risks associated with ARBs versus ACEi and Ang-II-stimulating versus Ang-II-inhibiting AHM-classes. Given the inevitability of potential confounding by indication in observational settings, a large-scale RCT is warranted to confirm whether treatment with these classes lowers dementia risk without increasing the risk of any other poor outcomes. However, the need for prolonged follow-up with dementia as an outcome, coupled with the associated high study costs, suggests that results from such a trial may be over a decade away. In the meantime, developing a framework for designing and analysing observational studies aimed at estimating the causal effects of interventions, ideally incorporating BP values, therapy adherence, and additional prescription pattern-influencing covariates, could further enhance our understanding of the relationship between AHM-classes and dementia risk.

## Contributors

EMvC was responsible for the initial conceptualisation, and data gathering of this study. Subsequently, all other authors, excluding MH, were involved in further refining the study design. JLS, JWvD, and EMvC had full access to the data of this study. MH curated data from the Utrecht GPRN. Statistical analyses, as well as the preparation of figures and tables, were conducted by JLS and JWvD, with statistical validation performed by WB and MHB. The first draft of the manuscript was written by JLS and JWvD, with input from all other authors during the review and editing process. The decision to submit the manuscript for publication was unanimously agreed upon by all authors. All named authors meet the criteria for authorship as outlined by the International Committee of Medical Journal Editors, take collective responsibility for the integrity of the work, and have provided their approval for its publication.

## Data sharing statement

All data used for this study have been collected from three individual Dutch General Practice Registration Networks and remain in their ownership. Coding scripts pertaining to the analyses can be obtained from the authors upon reasonable request.

## Declaration of interests

None of the authors declare competing interests.

## References

[1] Nichols E., Steinmetz J.D., Vollset S.E. (2022). Estimation of the global prevalence of dementia in 2019 and forecasted prevalence in 2050: an analysis for the global burden of disease study 2019. Lancet Public Health.

[2] Livingston G., Huntley J., Sommerlad A. (2020). Dementia prevention, intervention, and care: 2020 report of the lancet commission. Lancet.

[3] Lennon M.J., Makkar S.R., Crawford J.D., Sachdev P.S. (2019). Midlife hypertension and alzheimer's disease: a systematic review and meta-analysis. J Alzheimers Dis.

[4] Zhou B., Carrillo-Larco R.M., Danaei G. (2021). Worldwide trends in hypertension prevalence and progress in treatment and control from 1990 to 2019: a pooled analysis of 1201 population-representative studies with 104 million participants. Lancet.

[5] Hughes D., Judge C., Murphy R. (2020). Association of blood pressure lowering with incident dementia or cognitive impairment: a systematic review and meta-analysis. JAMA.

[6] Williamson J.D., Pajewski N.M., Auchus A.P. (2019). Effect of intensive vs standard blood pressure control on probable dementia: a randomized clinical trial. JAMA.

[7] den Brok M., van Dalen J.W., Abdulrahman H. (2021). Antihypertensive medication classes and the risk of dementia: a systematic review and network meta-analysis. J Am Med Dir Assoc.

[8] Unger T., Borghi C., Charchar F. (2020). 2020 International society of hypertension global hypertension practice guidelines. Hypertension.

[9] Williams B., Mancia G., Spiering W. (2018). 2018 ESC/ESH Guidelines for the management of arterial hypertension. Eur Heart J.

[10] Whelton P.K., Carey R.M., Aronow W.S. (2018). 2017 ACC/AHA/AAPA/ABC/ACPM/AGS/APhA/ASH/ASPC/NMA/PCNA guideline for the prevention, detection, evaluation, and management of high blood pressure in adults: executive summary: a report of the American college of cardiology/American heart association task force on clinical practice guidelines. Hypertension.

[11] Zhou B., Perel P., Mensah G.A., Ezzati M. (2021). Global epidemiology, health burden and effective interventions for elevated blood pressure and hypertension. Nat Rev Cardiol.

[12] Peters R., Yasar S., Anderson C.S. (2020). Investigation of antihypertensive class, dementia, and cognitive decline: a meta-analysis. Neurology.

[13] Ding J., Davis-Plourde K.L., Sedaghat S. (2020). Antihypertensive medications and risk for incident dementia and Alzheimer's disease: a meta-analysis of individual participant data from prospective cohort studies. Lancet Neurol.

[14] Schroevers J.L., Eggink E., Hoevenaar-Blom M.P. (2023). Antihypertensive medication classes and the risk of dementia over a decade of follow-up. J Hypertens.

[15] Richard E., Van den Heuvel E., Moll van Charante E.P. (2009). Prevention of dementia by intensive vascular care (PreDIVA): a cluster-randomized trial in progress. Alzheimer Dis Assoc Disord.

[16] Ojha U., Ruddaraju S., Sabapathy N. (2022). Current and emerging classes of pharmacological agents for the management of hypertension. Am J Cardiovasc Drugs.

[17] van Dalen J.W., Marcum Z.A., Gray S.L. (2021). Association of angiotensin II-stimulating antihypertensive use and dementia risk: post hoc analysis of the PreDIVA trial. Neurology.

[18] Marcum Z.A., Cohen J.B., Zhang C. (2022). Association of antihypertensives that stimulate vs inhibit types 2 and 4 angiotensin II receptors with cognitive impairment. JAMA Netw Open.

[19] Marcum Z.A., Gabriel N., Bress A.P., Hernandez I. (2023). Association of new use of antihypertensives that stimulate vs inhibit type 2 and 4 angiotensin II receptors with dementia among medicare beneficiaries. JAMA Netw Open.

[20] Overbeek J.A., Swart K.M.A., Houben E., Penning-van Beest F.J.A., Herings R.M.C. (2023). Completeness and representativeness of the pharmo general practitioner (GP) data: a comparison with national statistics. Clin Epidemiol.

[21] Austin P.C., Lee D.S., Fine J.P. (2016). Introduction to the analysis of survival data in the presence of competing risks. Circulation.

[22] Therneau T. Survival analysis [R package survival version 3.5-5]. https://CRAN.R-project.org/package=survival.

[23] Zhang Z., Reinikainen J., Adeleke K.A., Pieterse M.E., Groothuis-Oudshoorn C.G.M. (2018). Time-varying covariates and coefficients in Cox regression models. Ann Transl Med.

[24] White M.C., Fleeman R., Arnold A.C. (2019). Sex differences in the metabolic effects of the renin-angiotensin system. Biol Sex Differ.

[25] (2019). Cardiovasculair risicomanagement | NHG richtlijn M84. https://richtlijnen.nhg.org/standaarden/cardiovasculair-risicomanagement.

[26] Austin P.C., Fine J.P. (2017). Practical recommendations for reporting Fine-Gray model analyses for competing risk data. Stat Med.

[27] Wolkewitz M., Cooper B.S., Bonten M.J., Barnett A.G., Schumacher M. (2014). Interpreting and comparing risks in the presence of competing events. BMJ.

[28] Austin P.C., Latouche A., Fine J.P. (2020). A review of the use of time-varying covariates in the Fine-Gray subdistribution hazard competing risk regression model. Stat Med.

[29] Team RC (2020).

[30] Jellinger K.A. (2013). Pathology and pathogenesis of vascular cognitive impairment-a critical update. Front Aging Neurosci.

[31] Scotti L., Bassi L., Soranna D. (2021). Association between renin-angiotensin-aldosterone system inhibitors and risk of dementia: a meta-analysis. Pharmacol Res.

[32] Adesuyan M., Jani Y.H., Alsugeir D. (2022). Antihypertensive agents and incident alzheimer's disease: a systematic review and meta-analysis of observational studies. J Prev Alzheimers Dis.

[33] Bohlken J., Jacob L., Kostev K. (2019). The relationship between the use of antihypertensive drugs and the incidence of dementia in general practices in Germany. J Alzheimers Dis.

[34] Ferreira J.P., Gregson J., Böhm M., Rossignol P., Zannad F., Pocock S.J. (2021). Blood pressure reduction and anti-hypertensive treatment choice: a post-hoc analysis of the SPRINT trial. Clin Cardiol.

[35] Modesti P.A., Reboldi G., Cappuccio F.P. (2016). Panethnic differences in blood pressure in europe: a systematic review and meta-analysis. PLoS One.

[36] Leng B., Jin Y., Li G., Chen L., Jin N. (2015). Socioeconomic status and hypertension: a meta-analysis. J Hypertens.

[37] Hussain S., Singh A., Rahman S.O., Habib A., Najmi A.K. (2018). Calcium channel blocker use reduces incident dementia risk in elderly hypertensive patients: a meta-analysis of prospective studies. Neurosci Lett.

[38] Peters R., Schuchman M., Peters J., Carlson M.C., Yasar S. (2016). Relationship between antihypertensive medications and cognitive impairment: part II. Review of physiology and animal studies. Curr Hypertens Rep.

[39] Rouch L., Cestac P., Hanon O. (2015). Antihypertensive drugs, prevention of cognitive decline and dementia: a systematic review of observational studies, randomized controlled trials and meta-analyses, with discussion of potential mechanisms. CNS Drugs.

[40] Hajjar I., Brown L., Mack W.J., Chui H. (2012). Impact of Angiotensin receptor blockers on Alzheimer disease neuropathology in a large brain autopsy series. Arch Neurol.

[41] van Bussel E.F., Richard E., Arts D.L. (2017). Dementia incidence trend over 1992-2014 in the Netherlands: analysis of primary care data. PLoS Med.

[42] van den Dungen C., Hoeymans N., van den Akker M. (2014). Do practice characteristics explain differences in morbidity estimates between electronic health record based general practice registration networks?. BMC Fam Pract.

[43] Kist J.M., Vos R.C., Mairuhu A.T.A. (2023). SCORE2 cardiovascular risk prediction models in an ethnic and socioeconomic diverse population in The Netherlands: an external validation study. eClinicalMedicine.

[44] Thomopoulos C., Parati G., Zanchetti A. (2018). Effects of blood pressure-lowering treatment on cardiovascular outcomes and mortality: 14 - effects of different classes of antihypertensive drugs in older and younger patients: overview and meta-analysis. J Hypertens.

