# Supplementary material for: Antihypertensive medication classes and risk of incident dementia in primary care patients: a longitudinal cohort study in the Netherlands
Source: Lancet Reg Health Eur. 2024 May 15;42:100927. doi: 10.1016/j.lanepe.2024.100927 (PMC11126814; doi:10.1016/j.lanepe.2024.100927)
Supplement: Supplementary Methods and Tables [file mmc1.docx]

**Online supplemental content to**

Antihypertensive medication classes and risk of incident dementia in primary care patients: a longitudinal cohort study in the Netherlands

| **Table of contents** |  |
| --- | --- |
| **Supplementary Methods 1.** ICPC codes used for identifying incident dementia from the GPRN databases | 4 |
| **Supplementary Methods 2.** A brief explanation of the role of the Dutch GP within Dutch health care | 4 |
| **Supplementary Methods 3.** Description of analytic models | 4 |
| **Supplementary Methods 4.** AHM-classes and corresponding anatomical therapeutic chemical (ATC) codes | 5 |
| **Supplementary Methods 5. C**rude models, and models adjusted only for sex and age | 6 |
| **Supplementary Figure 1.** Examples for alterations in sensitivity analysis 1 and 2 | 7 |
| **Supplementary Table 1.** Population characteristics and medication use for the combined population split per GPRN | 8 |
| **Supplementary Table 2.** Common AHM-class combinations at baseline and during last year of observation | 9 |
| **Supplementary Table 3.** Associations between use of AHM-(sub)classes and dementia and mortality | 10 |
| **Supplementary Table 4.** Comparing Model 2 and 3 for dementia, mortality, and dementia or mortality | 11 |
| **Supplementary Table 5-I.** Sensitivity analysis with prescriptions at censoring | 12 |
| **Supplementary Table 5-II.** Main analysis with prescriptions used during the final year of observation for comparison | 12 |
| **Supplementary Table 6-I.** Sensitivity analysis with observations not carried forward | 13 |
| **Supplementary Table 6-II.** Main analysis with observations carried, forward up to two years for comparison | 13 |
| **Supplementary Table 7.** Comparison of three methods of adjusting for clustering. | 14 |
| **Supplementary Table 8.** Sensitivity analysis with AHM-class exposure as a proportion of total AHM time use | 15 |
| **Supplementary Table 9-I.** Sensitivity analysis excluding dementia cases within 1 year of the last regimen change | 16 |
| **Supplementary Table 9-II.** Sensitivity analysis excluding dementia cases within 1 year of initiating AHM | 16 |
| **Supplementary Table 10.** Dementia in participants with- or without history of major cardiovascular disease | 17 |
| **Supplementary Table 11-I.** Subgroup analyses stratified for sex, and diabetes | 18 |
| **Supplementary Table 11-I.** Subgroup analyses stratified for cardiovascular disease and congestive heart failure | 19 |
| **Supplementary Table 12.** Subgroup analysis stratified for number of simultaneously used AHM-classes | 20 |
| **Supplementary Table 13.** Subgroup analysis stratified for baseline age-group | 21 |
| **Supplementary Table 14-I** Analysis for individuals with their first AHM prescription starting 2015 or later | 22 |
| **Supplementary Table 14-II.** Analysis for individuals with their first AHM prescription before 2015 | 22 |
| **Supplementary Table 15-I.** Subgroup analyses stratified for sex, and diabetes | 23 |
| **Supplementary Table 15-II.** Subgroup analyses stratified for cardiovascular disease, and congestive heart failure | 24 |
| **Supplementary Table 16.** Subgroup analysis stratified for number of simultaneously used AHM-classes | 25 |
| **Supplementary Table 17.** Subgroup analysis stratified for baseline age-group | 26 |
| **Supplementary Table 18.** Competing risk according sub-distribution hazards (Fine & Gray) | 27 |

**Supplementary Methods 1. ICPC codes used for identifying incident dementia from the GPRN databases**

Dementia was defined as P70 (senile dementia/Alzheimer disease), the only code within the ICPC for dementia. Another code slightly related is P20 (memory/concentration/orientation impairment), but it is nonspecific and was disregarded.

**Supplementary Methods 2. A brief explanation of the role of the Dutch GP within Dutch health care**

Referral to secondary care systems and diagnoses in GPs EHR: In Dutch Health Care, the GP is the gatekeeper of the system. All Dutch citizens need to be registered with a GP. The vast majority of health concerns, including management of hypertension and diabetes, are primarily handled by GPs. Patients cannot access secondary care systems (such as specialist clinics, hospitals, including emergency rooms) without a referral from their GP. Exceptions are emergencies, such as major accidents and life-threatening conditions (such as myocardial infarction and stroke). In these occasions, the patient is directly transported to a hospital, without need of prior need consultation with a GP. However, in all instances (i.e. after GPs referral, and after emergency hospital consultation/admission) the patients GP receives correspondence of this hospital consultation/admission, including made diagnoses, and changes to medication regiments.

Nursing homes: In the past decades, the number of nursing homes has drastically decreased in the Netherlands. Most older adults keep living at home for much longer aided by home care nurses and informal care by family members, friends, and neighbours. In these cases patients will still be under the care of a GP, along with hospital specialists and other health care workers, who all report to the GP. In some instances, for instance when suffering from invalidating comorbidities such as dementia or stroke, patients will be admitted to nursing homes. In some cases, a GP will remains the treating physician, in other cases care will be transferred to a dedicated nursing home physicians. In case of permanent admission to a nursing home away from a GPs care, the participant will be censored at that time, as they deregistered from the participating GPRN. It is unlikely that this affected dementia estimates, as the vast majority of dementia diagnoses will have been made prior to admission.

**Supplementary Methods 3. Description of analytic models**

Our analyses only included individuals who were actively using one or more of the compared antihypertensive categories. We aimed to compare associations for ARBs, beta blockers, CCBs, and diuretics to those for ACE-inhibitors as reference category, in order to have a stable reference point (i.e. the dementia risk for same antihypertensive type as reference for each analysis). In analyses wherein individuals could only use one of these medications at a time, this could be done using four dummy variables (0/1 coded variables) for each of these medication types. The resulting cox regression formula in R would be comparable to:

*coxph(Surv(Start_Time,Stop_Time,Outcome_Status)~ARB_use+BB_use+CCB_use+Diuretic_use+covariates+frailty(GPRNs))*.

Individuals with 0 for all of these dummy variables would then necessarily be ACE-inhibitor users, and therefore ACE-inhibitors would be the reference category. However, in our analyses, individuals can use multiple medications at a time, therefore, individuals with 1 on one or more of the dummy variables may still use ACE-inhibitors. Adding another dummy variable for ACE-inhibitor use would not solve this problem, because the reference category would then change to individuals with 0 for all of the dummy variables, resulting in a calculated ‘floating average dementia risk’ as reference, which would be different for each of the analyses. To reinstate ACE-inhibitor use as the stable reference category for these analyses, we included a second categorical variable, which comprised the total number of antihypertensive medications taken, with 1 (the minimum) as reference category. This resulted in an R formula comparable to:

*coxph(Surv(Start_Time,Stop_Time,Outcome_Status)~ARB_use+BB_use+CCB_use+Diuretic_use+Total_nr_of_AHM_categorical+covariates+frailty(GPRNs))*.

Thereby, individuals with 0 for ARBs, BBs, CCBs, and Diuretics, must take ACEi, and have the reference 1 value in the total AHM used variable. However, individuals using -for example- both diuretics and ACEi, would present the HR for diuretic use, adjusted for another medication that was concurrently taken, which was not ARB, BB, or CCBs, since these variables would all be 0, and thereby must be ACEi use. Thereby, the resulting HRs for ARBs, BBs, CCBs and diuretics effectively translate to the HRs for these medications compared to ACE inhibitors

**Supplementary Methods 4. AHM-classes and corresponding ATC codes**

| **AHM-class^a^** | **ATC codes^b^** |
| --- | --- |
| ACEi | C09A; C09B |
| ARB | C09C; C09D |
| Beta blocker | C07; C09BX02; C09BX04; C09DX05 |
| CCB | C07FB; C08; C09BB; C09BX01; C09BX03; C09BX04; C09DB; C09DX01; C09DX03; C09DX06; C09DX07; C09XA53 |
| - Dihydropyridine | - C07FB; C08CA; C08G; C09BB02; C09BB03; C09BB04; C09BB05; C09BB06; C09BB07; C09BB12; C09BB13; C09BX01; C09BX03; C09BX04; C09DB; C09DX01; C09DX03; C09DX06; C09DX07; C09XA53 |
| - Non-dihydropyridine | - C08DA; C08DB; C08E; C09BB10; |
| Diuretic | C02L; C03; C07B; C07C; C07D; C08G; C09BA; C09BX01; C09BX03; C09DA; C09DX01; C09DX03; C09DX06; C09DX07; C09XA52; C09XA54 |
| - Thiazide(like) | - C03A; C03BA; C03EA; C07B; C07C; C07D; C08G; C09BA; C09BX-1; C09BX04; C09DA; C09DX01; C09DX03; C09DX06; C09DX07; C09XA52; C09XA54 |
| - Loop | - C03CA; C03CB; C03EB |
| - Potassium sparing | - C03AB; C03BB; C03D; C03E |

^a^ Ang-II-stimulating AHM include all codes associated with ARB, dihydropyridine CCB, and Thiazide(like) diuretics. Ang-II-inhibiting AHM include all codes associated with ACEi, beta blocker, and non-dihydropyridine CCB ^b^ Includes all underlying codes (e.g. C09A includes, C09AA01- C09AA16).

AHM = antihypertensive medication; ATC = anatomical therapeutic chemical; ACEi = angiotensin-converting enzyme inhibitor; ARB = angiotensin receptor blocker, CCB = calcium channel blocker.

**Supplementary Methods 5. Results of crude models, and separate models adjusted only for sex and age, to evaluate the separate effects of adjusting for these factors on the results of model 1**

| AHM-class | Dementia cases/  total person years^a^  n(‰) | HR crude model (95%CI) | P-value | HR crude model + baseline age (95%CI) | P-value | HR crude model + sex (95%CI) | P-sex | HR Model 1 ^b^  (95%CI) | P-value |
| --- | --- | --- | --- | --- | --- | --- | --- | --- | --- |
| ACEi | 2251/357,122 (6·3) | *Reference* | | | | | | | |
| ARB | 1346/246,006 (5·5) | 0·84 (0·79-0·90) | <0·001 | 0·85 (0·79-0·91) | <0·001 | 0·82 (0·76-0·88) | <0·001 | 0·85 (0·79-0·91) | <0·001 |
| Beta blocker | 2989/509,093 (5·9) | 0·91 (0·85-0·98) | 0·01 | 0·79 (0·73-0·85) | <0·001 | 0·88 (0·82-0·94) | <0·001 | 0·79 (0·73-0·85) | <0·001 |
| CCB | 1759/300,174 (5·9) | 0·90 (0·83-0·98) | 0·01 | 0·76 (0·70-0·82) | <0·001 | 0·87 (0·80-0·94) | <0·001 | 0·76 (0·70-0·82) | <0·001 |
| Diuretic | 3167/533,719 (5·9) | 0·94 (0·87-1·02) | 0·11 | 0·64 (0·59-0·69) | <0·001 | 0·87 (0·80-0·94) | <0·001 | 0·64 (0·59-0·69) | <0·001 |

^a^ Within class of interest ^b^ Adjusted for baseline age, sex & AHM-classes used simultaneously. Individual participants can be represented in multiple AHM-classes in case of combination therapy and medication switching over time.

AHM = antihypertensive medication; HR = hazard ratio; CI = confidence interval; ACEi = angiotensin-converting enzyme inhibitor, ARB = angiotensin receptor blocker, CCB = calcium channel blocker.

**Supplementary Figure 1. Examples for alterations done in sensitivity analysis 1 and 2
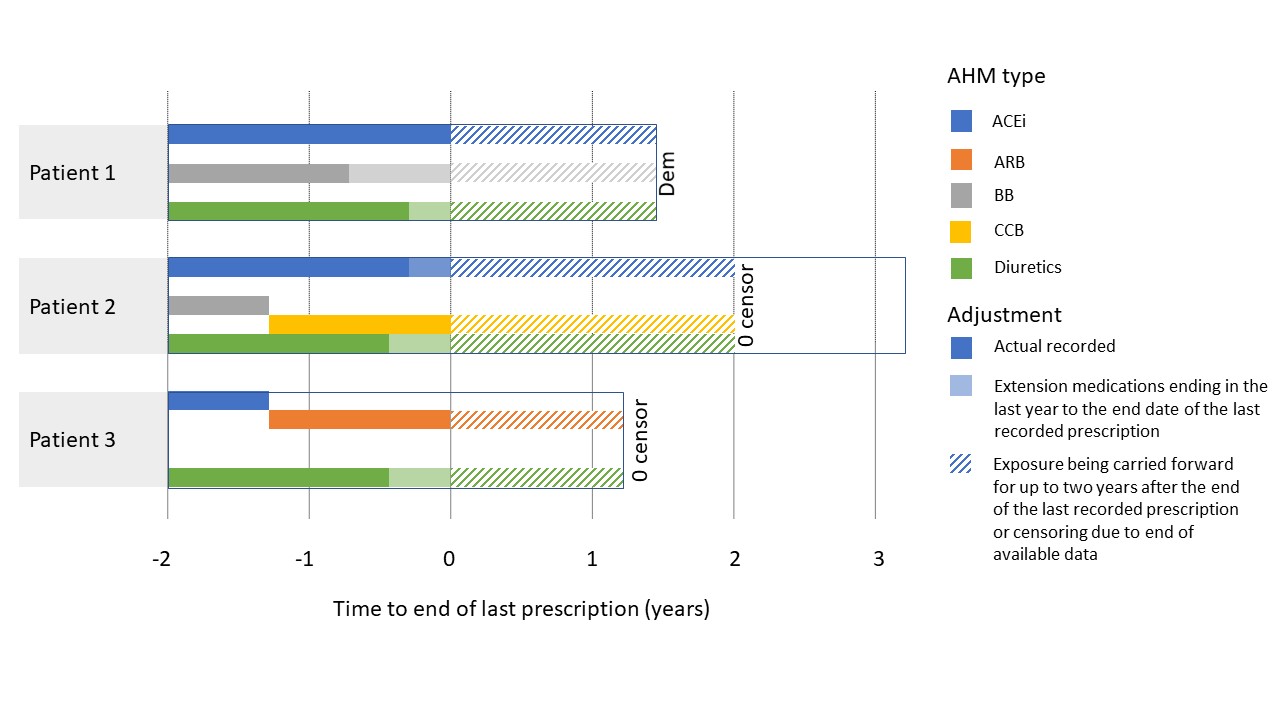
**

Dem = dementia; 0 censor = censored without dementia diagnosis during the observed period. AHM = antihypertensive medication; HR = hazard ratio; CI = confidence interval; ACEi = angiotensin-converting enzyme inhibitor; ARB = angiotensin receptor blocker, BB = beta blockers CCB = calcium channel blocker.

*Reading example: For patient 1, ACEi (blue) was the last ending prescription. BB (grey) and Diuretics (green) ended within one year before, so their use was extended to the end of ACEi (light shaded grey and green). These three prescription exposures were then carried forward for up to 2 years (dashed shade), in this case to 1.4 years when the patient was censored with dementia. For patient 2, CCB (yellow) was the last ending prescription. ACEi (blue) and Diuretics (green) ended within one year before, and were therefore extended up to the end of CCBs (light shaded blue and green). BB (grey) ended more than a year before CCBs (yellow), and was therefore not extended. For ACEi, CCBs and Diuretics, prescription exposures were carried forward for 2 years (dashed blue, yellow and green), at which time no event had occurred, so the patient was censored as no dementia. For patient 3, ARB (orange) was the last ending prescription. Only diuretics (green) ended in the last year before that, so was extended up to ARB’s end (light shaded green). The last prescriptions were then carried forward for up to 2 years (dashed orange and green). In this case, the patient was censored without an event after 1.2 years, because that was the time up until which information was available for this patient.*

**Supplementary Table 1. Population characteristics and medication use for the combined population split per GPRN**

|  | **AMC** | **UMCU** | **VUMC** | **Combined** |
| --- | --- | --- | --- | --- |
| ***Baseline*** | N=40,207 | N=49,167 | N=43,981 | N=133,355 |
| Women, n(%) | 20,890 (52·0) | 27,413 (55·8) | 24,581 (55·9) | 72,884 (54·7) |
| Baseline age, median[IQR] | 68·2 [62·9-75·5] | 68·0 [61·1-75·6] | 68·6 [61·9-76·3] | 68·2 [62·0-75·8] |
| History of type 2 diabetes, n(%) | 10,869 (27·0) | 7866 (16·0) | 7920 (18·0) | 26,655 (20·0) |
| History of coronary heart disease, n(%) | 5141 (12·8) | 4419 (9·0) | 4233 (9·6) | 13,793 (10·3) |
| History of stroke, n(%) | 3783 (9·3) | 2419 (6·0) | 2873 (6·5) | 9075 (6·8) |
| ACEi users at baseline | 11,822 (29·4) | 11,821 (29·4) | 10,902 (24·8) | 34,545 (25·9) |
| ARB users at baseline, n(%) | 6251 (15·5) | 5020 (12·5) | 6398 (14·5) | 17,669 (13·2) |
| Beta blocker users at baseline, n(%) | 16,121 (40·0) | 16,929 (42·0) | 17,235 (39·2) | 50,285 (37·7) |
| CCB users at baseline, n(%) | 9785 (24·3) | 6089 (15·1) | 7986 (18·2) | 23,860 (17·9) |
| Diuretic users at baseline, n(%) | 16,813 (41·8) | 17,852 (44·3) | 16,411 (37·3) | 51,076 (38·3) |
| ***Follow-up*** |  |  |  |  |
| Median observed years [IQR] | 7·6 [5·2-10·4] | 11·8 [7·0-17·0] | 9·4 [5·9-15·7] | 7·6 [4·1-11·0] |
| Total observed person years | 248,973 | 407,877 | 347,926 | 1,004,775 |
| Median censoring age [IQR] | 74·9 [69·6-81·8] | 78·7 [71·5-84·6] | 78·0 [71·1-84·1] | 76·5 [70·7-83·6] |
| Incident type 2 diabetes, n(%) | 1534 (3·8) | 4967 (9·6) | 3727 (8·5) | 9,958 (7·5) |
| Incident coronary heart disease, n(%) | 1029 (2·6) | 2290 (4·7) | 2027 (4·6) | 5346 (4·0) |
| Incident stroke, n(%) | 516 (1·3) | 1237 (2·5) | 1268 (2·9) | 3021 (2·3) |
| Incident dementia, n(%) | 1527 (3·8) | 2179 (5·4) | 2171 (4·9) | 5877 (4·4) |
| Mortality, n(%) | 4588 (11·4) | 5076 (12·6) | 4413 (10·0) | 14,079 (10·6) |
| ACEi users at censoring, n(%)* | 14,663 (36·4) | 16,618 (41·3) | 15,081 (34·3) | 46,362 (34·8) |
| ARB users at censoring, n(%)* | 9291 (23·1) | 10,142 (25·2) | 11,033 (25·1) | 30,466 (22·8) |
| Beta blocker users at censoring, n(%)* | 19,930 (49·5) | 21,328 (53·0) | 21,955 (49·9) | 63,213 (47·4) |
| CCB users at censoring, n(%)* | 15,236 (37·9) | 12,986 (32·2) | 14,117 (32·1) | 42,339 (31·7) |
| Diuretic users at censoring, n(%)* | 20,324 (50·5) | 24,636 (61·2) | 22,651 (51·5) | 67,611 (50·7) |

* AHM use at censoring was defined as used during the last year of observation before reaching an endpoint.
GPRN = General Practice Registration Network; AMC = Academic Medical Center; UMCU = University Medical Center Utrecht; VUMC = Vrije Universiteit Medical Center; ACEi = angiotensin-converting enzyme inhibitor; ARB = angiotensin receptor blocker; CCB = calcium channel blocker; AHM = antihypertensive medication

**Supplementary Table 2. Common AHM-class combinations at baseline and during last year of follow-up**

| **AHM-class combinations at baseline** | **N (%)**  n=133,355 |
| --- | --- |
| Beta blocker monotherapy | 31694 (23·8) |
| Diuretic monotherapy | 28,524 (21·4) |
| ACEi monotherapy | 19,174 (14·4) |
| CCB monotherapy | 13,133 (9·8) |
| ARB monotherapy | 8039 (6·0) |
| ACEi+diuretic | 4824 (3·6) |
| Beta blocker+diuretic | 4462 (3·3) |
| ARB+diuretic | 3742 (2·8) |
| ACEi+beta blocker | 3576 (2·7) |
| ACEi+beta blocker+diuretic | 2541 (1·9) |
| **AHM-class combinations at during last year of follow-up** | **N (%)**  n=133,355 |
| Beta blocker monotherapy | 17,300 (13·0) |
| Diuretic monotherapy | 15,210 (11·4) |
| ACEi monotherapy | 11,219 (8·4) |
| CCB monotherapy | 9010 (6·8) |
| Beta blocker+diuretic | 7814 (5·9) |
| ACEi+diuretic | 7354 (5·5) |
| ACEi+beta blocker+diuretic | 7195 (5·4) |
| ARB monotherapy | 5611 (4·2) |
| ACEi+beta blocker | 5542 (4·2) |
| ARB+diuretic | 5268 (4·0) |

This table depicts the most common combinations of simultaneously used AHM-classes, including monotherapy. The top section depicts combinations at baseline (i.e. when participants enter the GPRN). The bottom depicts combinations during the last year of observation (which was the exposure for our main analyses). AHM = antihypertensive medication; ACEi = angiotensin-converting enzyme inhibitor; ARB = angiotensin receptor blocker; CCB = calcium channel blocker.

**Supplementary Table 3. Associations between use of AHM-(sub)classes and dementia and mortality, compared with use of ACEi**

| AHM-(sub)class | Dementia cases/  total person years^a^ n(‰) | HR Dementia^b^ (95%CI) | P-value Dementia | Mortality cases/  total person years^a^ n(‰) | HR Mortality^b^ (95%CI) | P-value Mortality |
| --- | --- | --- | --- | --- | --- | --- |
| ACEi | 2251/357,122 (6·3) | *Reference* | | 5140/361,994 (14·2) | *Reference* | |
| ARB | 1346/246,006 (5·5) | 0·85 (0·79-0·91) | <0·001 | 2946/248,697 (11·9) | 0·88 (0·84-0·92) | <0·001 |
| Beta blocker | 2989/509,093 (5·9) | 0·81 (0·76-0·88) | <0·001 | 7852/515,410 (15·2) | 1·06 (1·01-1·12) | 0·015 |
| CCB |  |  |  |  |  |  |
| - Dihydropyridine | 1511/257,851 (5·9) | 0·79 (0·72-0·86) | <0·001 | 3681/261,284 (14·1) | 1·07 (1·02-1·14) | 0·009 |
| - Non-dihydropyridine | 257/40,308 (6·4) | 0·80 (0·70-0·91) | 0·001 | 741/40,954 (18·1) | 1·13 (1·04-1·23) | 0·004 |
| Diuretic |  |  |  |  |  |  |
| - Thiazide(like) | 1908/389,315 (4·9) | 0·75 (0·69-0·82) | <0·001 | 3420/393,295 (8·7) | 0·87 (0·83-0·93) | <0·001 |
| - Loop | 1374/129,170 (10·6) | 0·78 (0·71-0·85) | <0·001 | 6670/132,748 (50·3) | 3·05 (2·89-3·22) | <0·001 |
| - Potassium sparing | 572/78,464 (7·3) | 0·77 (0·69-0·86) | <0·001 | 2631/79,687 (33·0) | 1·50 (1·42-1·59) | <0·001 |

^a^ Within class of interest ^b^ Model 2, adjusted for baseline age, sex, number of AHM-classes simultaneously used, type 2 diabetes, myocardial infarction and stroke as time dependent variables. Individual participants may be represented in multiple AHM-classes in case of combination therapy and medication switching over time.

AHM = antihypertensive medication; HR = hazard ratio; CI = confidence interval; ACEi = angiotensin-converting enzyme inhibitor; ARB = angiotensin receptor blocker, CCB = calcium channel blocker.

Note: point estimates for ACEi, ARBs, and beta blockers are slightly different from Table 2 in the main text because the models here include all the subtypes of CCBs and diuretics separately, instead of for CCBs and Diuretics combined as in the main analyses. The slightly different predictors in the models resulted in slightly different results for ACEi, ARBs and beta blockers.

**Supplementary Table 4. Comparing Model 2 and 3 for dementia, mortality, and dementia or mortality**

|  | Dementia | | | Mortality | | | Dementia or Mortality | | |
| --- | --- | --- | --- | --- | --- | --- | --- | --- | --- |
| AHM-class | **Cases/PY n (‰)** | **HR model 2**  **(95%CI)** | **HR model 3**  **(95%CI)** | **Cases/PY n (‰)** | **HR model 2**  **(95%CI)** | **HR model 3**  **(95%CI)** | **Cases/PY n (‰)** | **HR model 2**  **(95%CI)** | **HR model 3**  **(95%CI)** |
| ACEi | 2,251/357,122 (6·3) | *Reference* | | 5,140/361,994 (14·2) | *Reference* | | 7662/357,123  (21·5) | *Reference* | |
| ARB | 1,346/246,006 (5·5) | 0·86  (0·80-0·92) | 0·86  (0·80-0·92) | 2,946/248,697 (11·9) | 0·83  (0·80-0·87) | 0·85  (0·81-0·89) | 4443/246,006  (18·1) | 0·85  (0·81-0·88) | 0·86  (0·83-0·90) |
| Beta blocker | 2,989/509,093 (5·9) | 0·81  (0·75-0·87) | 0·81  (0·75-0·87) | 7,852/515,410 (15·2) | 1·21  (1·15-1·27) | 1·16  (1·11-1·22) | 11,225/509,093  (22·1) | 1·06  (1·02-1·11) | 1·04  (0·99-1·08) |
| CCB | 1,759/300,174 (5·9) | 0·77  (0·71-0·84) | 0·77  (0·71-0·84) | 4,357/304,486 (14·3) | 1·04  (0·99-1·10) | 1·10  (1·04-1·16) | 6272/300,174  (20·9) | 0·96  (0·92-1·00) | 0·99  (0·95-1·04) |
| Diuretic | 3,167/533,719 (5·9) | 0·65  (0·61-0·70) | 0·65  (0·60-0·70) | 9,526/541,233 (17·6) | 1·69  (1·60-1·78) | 1·47  (1·30-1·55) | 13,157/533,719  (24·7) | 1·26  (1·21-1·31) | 1·16  (1·11-1·21) |
|  |  |  |  |  |  |  |  |  |  |
| Ang-II-inhibiting | 4312/727,829  (5·9) | *Reference* | | 10,409/738,051 (14·1) | *Reference* | | 14,290/727,691 (19·6) | *Reference* | |
| Ang-II-stimulating | 3491/631,650 (5·5) | 0·88  (0·82-0·95) | 0·88  (0·82-0·95) | 7330/639,836 (11·5) | 0·86  (0·82-0·91) | 0·89  (0·85-0·93) | 10,511/631,568 (16·6) | 0·87  (0·84-0·91) | 0·89  (0·85-0·93) |

Hazard ratio’s (HR) for incident outcomes according Cox regression with time varying covariates· HRs present model 2, adjusting for age and sex at baseline, and number of AHM-classes simultaneously used, type 2 diabetes, myocardial infarction, and stroke as time dependent variables. Model 3 is additionally adjusted for congestive heart failure. Analyses for Ang-II inhibiting versus stimulating AHM were additionally adjusted for K-sparing & Loop diuretics as both subclasses are not represented in either Ang-II-stimulating or Ang-II-inhibiting AHM. Ang-II-inhibiting AHM include: ACEi, Beta blocker & non-dihydropyridine CCB ; AngII-stimulating AHM: ARB, dihydropyridine CCB & Thiazide(like) diuretics. We found no evidence for non-proportionality in our Cox models according to the distribution of the Schoenfeld residuals. Cases/PY represent the total number of incident cases (Cases) that occurred during the total person years (PY) of exposure observed for each class of interest. Individual participants may be represented in multiple AHM-classes in case of combination therapy and medication switching over time.

Abbreviations: AHM = antihypertensive medication; HR = hazard ratio; CI = confidence interval; ACEi = angiotensin-converting enzyme inhibitor; ARB = angiotensin receptor blocker, CCB = calcium channel blocker; Ang-II = angiotensin II type 2 receptor

**Supplementary Table 5-I. Sensitivity analysis with prescriptions at censoring. Association between AHM-classes and dementia, compared to use of ACEi**

| AHM-class | Dementia cases/total person years^a^  n(‰) | HR^b^ (95%CI) | P-value |
| --- | --- | --- | --- |
| ACEi | 1867/350,717 (5·3) | *Reference* | |
| ARB | 1098/502,026 (4·5) | 0·89 (0·82-0·96) | <0·002 |
| Beta blocker | 2476/502,026 (4·9) | 0·80 (0·75-0·86) | <0·001 |
| CCB | 1417/293,396 (4·8) | 0·84 (0·78-0·91) | <0·001 |
| Diuretic | 2530/524,831 (4·8) | 0·66 (0·61-0·71) | <0·001 |

**Supplementary Table 5-II. Main analysis with prescriptions used during the final year of observation for comparison. Association between AHM-classes and dementia, compared to use of ACEi.**

| AHM-class | Dementia cases/total person years^a^  n(‰) | HR^b^ (95%CI) | P-value |
| --- | --- | --- | --- |
| ACEi | 2251/357,122 (6·3) | *Reference* | |
| ARB | 1346/246,006 (5·5) | 0·87 (0·81-0·93) | <0·001 |
| Beta blocker | 2989/509,093 (5·9) | 0·81 (0·76-0·88) | <0·001 |
| CCB | 1759/300,174 (5·9) | 0·78 (0·72-0·85) | <0·001 |
| Diuretic | 3167/533,719 (5·9) | 0·67 (0·62-0·72) | <0·001 |

^a^ Within class of interest ^b^ Model 2, adjusted for baseline age, sex, number of AHM-classes used simultaneously, type 2 diabetes, myocardial infarction and stroke as time dependent variables. Individual participants may be represented in multiple AHM-classes in case of combination therapy and medication switching over time.

AHM= antihypertensive medication; HR = hazard ratio; CI = confidence interval; ACEi = angiotensin-converting enzyme inhibitor; ARB = angiotensin receptor blocker, CCB = calcium channel blocker

**Supplementary Table 6-I. Sensitivity analysis with observations not carried forward. Association between AHM-classes and dementia, compared to use of ACEi**

| AHM-class | Dementia cases/total person years^a^  n(‰) | HR^b^ (95%CI) | P-value |
| --- | --- | --- | --- |
| ACEi | 1468/347,487 (4·2) | R*eference* | |
| ARB | 915/240,795 (3·8) | 0·88 (0·81-0·96) | 0·006 |
| Beta blocker | 2015/492,799 (4·1) | 0·84 (0·76-0·92) | <0·001 |
| CCB | 1156/292,527 (4·0) | 0·73 (0·66-0·81) | <0·001 |
| Diuretic | 2059/517,569 (4·0) | 0·64 (0·58-0·71) | <0·001 |

**Supplementary Table 6-II. Main analysis with observations carried forward up to two years for comparison. Association between AHM-classes and dementia, compared to use of ACEi.**

| AHM-class | Dementia cases/total person years^a^  n(‰) | HR^b^ (95%CI) | P-value |
| --- | --- | --- | --- |
| ACEi | 2251/357,122 (6·3) | *Reference* | |
| ARB | 1346/246,006 (5·5) | 0·87 (0·81-0·93) | <0·001 |
| Beta blocker | 2989/509,093 (5·9) | 0·81 (0·76-0·88) | <0·001 |
| CCB | 1759/300,174 (5·9) | 0·78 (0·72-0·85) | <0·001 |
| Diuretic | 3167/533,719 (5·9) | 0·67 (0·62-0·72) | <0·001 |

^a^ Within class of interest ^b^ Model 2, adjusted for baseline age, sex, number of AHM-classes used simultaneously, type 2 diabetes, myocardial infarction and stroke as time dependent variables. Individual participants may be represented in multiple AHM-classes in case of combination therapy and medication switching over time.

AHM= antihypertensive medication; HR = hazard ratio; CI = confidence interval; ACEi = angiotensin-converting enzyme inhibitor; ARB = angiotensin receptor blocker, CCB = calcium channel blocker

**Supplementary Table 7. Comparison of three methods of adjusting for clustering. Associations between AHM-classes and dementia**

|  |  | **Random terms for general practices** | **Fixed terms for datasets** | **Main model (random terms for datasets)** |
| --- | --- | --- | --- | --- |
| **AHM-class** | **Cases/PY n (‰)** | **HR^a^** | **HR^a^** | **HR^a^** |
| ACEi | 2,251/357,122 (6·3) | *Reference* | *Reference* | *Reference* |
| ARB | 1,346/246,006 (5·5) | 0·86 (0·80-0·92) | 0·86 (0·80-0·92) | 0·86 (0·80-0·87) |
| Beta blocker | 2,989/509,093 (5·9) | 0·78 (0·72-0·84) | 0·81 (0·75-0·87) | 0·71 (0·75-0·87) |
| CCB | 1,759/300,174 (5·9) | 0·79 (0·73-0·86) | 0·77 (0·71-0·84) | 0·77 (0·71-0·84) |
| Diuretic | 3,167/533,719 (5·9) | 0·64 (0·59-0·69) | 0·65 (0·60-0·70) | 0·65 (0·60-0·70) |

This study includes data from 79 individual general practices spread over 3 general practice registration networks. Hazard ratio’s (HR) for incident dementia according Cox regression with time varying covariates. ^a^ Model 2, adjusting for age and sex at baseline, and number of AHM-classes simultaneously used, type 2 diabetes, myocardial infarction, and stroke as time dependent variables. Abbreviations: AHM = antihypertensive medication; HR = hazard ratio; CI = confidence interval; ACEi = angiotensin-converting enzyme inhibitor; ARB = angiotensin receptor blocker, CCB = calcium channel blocker.

**Supplementary Table 8. Sensitivity analysis with AHM-class exposure as a proportion of total AHM time use**

|  |  |  | Model 1^c^ | | Model 2^d^ | |
| --- | --- | --- | --- | --- | --- | --- |
| Proportion cut-off^a^ | **AHM-class** | **Dementia cases/total person years^b^ n(‰)** | **HR (95%CI)** | **P-value** | **HR (95%CI)** | **P-value** |
| >25% | ACEi | 2403/380,859 (6·3) | *Reference* | | *Reference* | |
|  | ARB | 1489/255,152 (5·8) | 0·88 (0·82-0·95) | <0·001 | 0·90 (0·84-0·97) | 0·003 |
|  | Beta blocker | 3295/533,674 (6·2) | 0·86 (0·80-0·92) | <0·001 | 0·89 (0·83-0·96) | 0·003 |
|  | CCB | 1919/308,169 (6·2) | 0·79 (0·73-0·85) | <0·001 | 0·82 (0·76-0·89) | <0·001 |
|  | Diuretic | 3513/561,935 (6·3) | 0·69 (0·64-0·75) | <0·001 | 0·73 (0·67-0·79) | <0·001 |
| >50% | ACEi | 2051/339,164 (6·1) | *Reference* | | *Reference* | |
|  | ARB | 1268/224,698 (5·6) | 0·90 (0·83-0·96) | 0·003 | 0·92 (0·85-0·98) | 0·02 |
|  | Beta blocker | 2970/494,116 (6·0) | 0·87 (0·81-0·93) | <0·001 | 0·90 (0·84-0·97) | 0·006 |
|  | CCB | 1567/264,334 (5·9) | 0·80 (0·73-0·86) | <0·001 | 0·83 (0·76-0·90) | <0·001 |
|  | Diuretic | 3103/514,171(6·0) | 0·71 (0·65-0·76) | <0·001 | 0·74 (0·68-0·80) | <0·001 |
| >75% | ACEi | 1755/294,456 (6·0) | *Reference* | | *Reference* | |
|  | ARB | 1028/187,498 (5·5) | 0·89 (0·82-0·96) | 0·002 | 0·90 (0·83-0·98) | 0·01 |
|  | Beta blocker | 2580/447,649 (5·8) | 0·84 (0·78-0·90) | <0·001 | 0·87 (0·81-0·94) | <0·001 |
|  | CCB | 1238/216,397 (5·7) | 0·78 (0·72-0·85) | <0·001 | 0·81 (0·75-0·89) | <0·001 |
|  | Diuretic | 2638/451,444 (5·8) | 0·69 (0·64-0·75) | <0·001 | 0·72 (0·67-0·78) | <0·001 |

^a^ Proportion of time class of interest was used divided by total time any AHM was used. ^b^ Within class of interest ^c^ Adjusted for baseline age, sex and number of AHM-classes used simultaneously ^d^ and type 2 diabetes, myocardial infarction and stroke as time dependent variables. Individual participants may be represented in multiple AHM-classes in case of combination therapy and medication switching over time.

AHM = antihypertensive medication; HR = hazard ratio; CI = confidence interval; ACEi = angiotensin-converting enzyme inhibitor; ARB = angiotensin receptor blocker, CCB = calcium channel blocker.

**Supplementary Table 9-I. Sensitivity analysis excluding dementia cases within 1 year of the last regimen change**

| **AHM-class** | **Cases/PY n (‰)** | **HR^a^ (95%CI)** | **P-value** |
| --- | --- | --- | --- |
| ACEi | 2020/356,971 (5·7) | *Reference* | |
| ARB | 1240/245,917 (5·0) | 0·87 (0·81-0·93) | <0·001 |
| Beta blocker | 2698/508,831 (5·3) | 0·81 (0·75-0·87) | <0·001 |
| CCB | 1605/300,087 (5·5) | 0·77 (0·71-0·84) | <0·001 |
| Diuretic | 2828/53,495 (5·3) | 0·64 (0.59-0·69) | <0·001 |

^a^ Model 2 , adjusting for age and sex at baseline, and number of AHM-classes simultaneously used, type 2 diabetes, myocardial infarction, and stroke as time-dependent variables. Abbreviations: AHM = antihypertensive medication; HR = hazard ratio; CI = confidence interval; ACEi = angiotensin-converting enzyme inhibitor; ARB = angiotensin receptor blocker, CCB = calcium channel blocker.

**Supplementary Table 9-II. Sensitivity analysis excluding dementia cases within 1 year of initiating AHM**

| **AHM-class** | **Cases/PY n (‰)** | **HR^a^ (95%CI)** | **P-value** |
| --- | --- | --- | --- |
| ACEi | 1645/356,274 (4·6) | *Reference* | |
| ARB | 1014/245,515 (4·1) | 0·86 (0·80-0·93) | <0·001 |
| Beta blocker | 2150/507,904 (4·2) | 0·80 (0·74-0·87) | <0·001 |
| CCB | 1317/299,546 (4·4) | 0·79 (0·72-0·85) | <0·001 |
| Diuretic | 2245/532,464 (4·2) | 0·63 (0·59-0·69) | <0·001 |

^a^ Model 2 , adjusting for age and sex at baseline, and number of AHM-classes simultaneously used, type 2 diabetes, myocardial infarction, and stroke as time-dependent variables. Abbreviations: AHM = antihypertensive medication; HR = hazard ratio; CI = confidence interval; ACEi = angiotensin-converting enzyme inhibitor; ARB = angiotensin receptor blocker, CCB = calcium channel blocker.

**Supplementary Table 10. Dementia in participants with- or without history of major cardiovascular disease**

|  | **Dementia in participants with history of major CVD**  **N=1387** | | **Dementia in participants without history of major CVD**  **N=4490** | | **Dementia in all participants**  **N=5877** | |
| --- | --- | --- | --- | --- | --- | --- |
| **AHM-class** | **Cases/total PY, n(‰)** | **HR^a^ (95%CI)** | **Cases/total PY, n(‰)** | **HR^a^ (95%CI)** | **Cases/total PY, n(‰)** | **HR^a^ (95%CI)** |
| ACEi | 592/857,053 (0·68) | *Reference* | 1659/857,053 (1·90) | *Reference* | 2251/857,053 (2·57) | *Reference* |
| ARB | 306/589,285 (0·52) | 0·76 (0·66-0·88) | 1040/589,285 (1·76) | 0·89 (0·83-0·97) | 1346/589,285 (2·28) | 0·86 (0·80-0·92) |
| Beta blocker | 861/1,248,816 (0·69) | 1·05 (0·90-1·23) | 2128/1,248,816 (1·70) | 0·75 (0·69-0·82) | 2989/1,248,816 (2·39) | 0·81 (0·75-0·87) |
| CCB | 413/731,855 (0·56) | 0·66 (0·56-0·78) | 1346/731,855 (1·84) | 0·82 (0·75-0·90) | 1758/731,855 (2·40) | 0·77 (0·72-0·84) |
| Diuretic | 861/1,285,692 (0·55) | 0·49 (0·42-0·58) | 2463/1,285,692 (1·92) | 0·70 (0·64-0·77) | 3167/1,285,692 (2·92) | 0·65 (0·60-0·70) |

Dementia in participants with/without history of major CVD (i.e. stroke or myocardial infarction), and dementia in all participants, regardless history of CVD. Hazard ratio’s (HR) for incident outcomes according Cox regression with time varying covariates. ^a^ adjusted for baseline age and sex, and number of AHM-classes simultaneously used, type 2 diabetes, and congestive heart failure as time dependent variables. Abbreviations: CVD = cardiovascular disease; AHM = antihypertensive medication; PY = person years; HR = hazard ratio; CI = confidence interval; ACEi = angiotensin-converting enzyme inhibitor; ARB = angiotensin receptor blocker, CCB = calcium channel blocker.

**Supplementary Table 11-I. Subgroup analyses stratified for sex, and diabetes; associations between use of AHM-classes and dementia, compared to use of ACEi**

| Subgroup | AHM-class | Dementia cases/total person years^a^ n(‰) | HR^b^ (95%CI) | P-value interaction  (men vs women) |
| --- | --- | --- | --- | --- |
| Women | ACEi | 1246/168,312 (7·4) | *Ref* |  |
|  | ARB | 889/139,116 (6·4) | 0·87 (0·80-0·95) | *Ref* |
|  | Beta blocker | 1864/280,454 (6·6) | 0·81 (0·74-0·88) | *Ref* |
|  | CCB | 1139/159,725 (7·1) | 0·80 (0·73-0·87) | *Ref* |
|  | Diuretic | 2111/321,857 (6·6) | 0·64 (0·68-0·69) | *Ref* |
| Men | ACEi | 1005/188,811 (5·3) | *Ref* |  |
|  | ARB | 457/106,890 (4·3) | 0·83 (0·75-0·93) | 0·46 |
|  | Beta blocker | 1125/228,639 (4·9) | 0·80 (0·73-0·89) | 0·94 |
|  | CCB | 620/140,449 (4·4) | 0·73 (0·65-0·81) | 0·13 |
|  | Diuretic | 1056/211,862 (5·0) | 0·69 (0·62-0·76) | 0·17 |
| Subgroup | **AHM-class** | **Dementia cases/total person years^a^ n(‰)** | **HR^b^ (95%CI)** | **P-value interaction**  **(diabetes vs no diabetes)** |
| No diabetes | ACEi | 1426/227,505 (6·3) | *Ref* |  |
|  | ARB | 890/165,268 (5·4) | 0·81 (0·73-0·91) | *Ref* |
|  | Beta blocker | 2058/366,613 (5·6) | 0·80 (0·73-0·92) | *Ref* |
|  | CCB | 1165/203,860 (5·7) | 0·76 (0·68-0·86) | *Ref* |
|  | Diuretic | 2141/366,761 (5·8) | 0·66 (0·59-0·74) | *Ref* |
| Diabetes | ACEi | 825/129,618 (6·4) | *Ref* |  |
|  | ARB | 456/80,738 (5·7) | 0·89 (0·81-0·96) | 0·22 |
|  | Beta blocker | 913/142,480 (6·5) | 0·81 (0·75-0·88) | 0·84 |
|  | CCB | 594/96,314 (6·2) | 0·78 (0·71-0·85) | 0·71 |
|  | Diuretic | 1026/166,958 (6·2) | 0·65 (0·60-0·71) | 0·75 |

^a^ Within class of interest ^b^ Model 2, adjusted for baseline age, sex, number of AHM-classes simultaneously used, type 2 diabetes, myocardial infarction and stroke as time dependent variables. Individual participants may be represented in multiple AHM-classes in case of combination therapy and medication switching over time. Similarly, patients may switch subgroup after developing diabetes, CVD or congestive heart failure over time. AHM = antihypertensive medication; HR = hazard ratio; CI = confidence interval; P-int = p for interaction between the two subgroups; CVD = cardiovascular disease; CHF = congestive heart failure; ACEi = angiotensin-converting enzyme inhibitor; ARB = angiotensin receptor blocker; CCB = calcium channel blocker

**Supplementary Table 11-II. Subgroup analyses stratified for cardiovascular disease** ^a^ **and congestive heart failure; association between use of AHM-classes and dementia, compared to use of ACEi**

| Subgroup | AHM-class | Dementia cases/total person years^b^ n(‰) | HR^c^ (95%CI) | P-value interaction  (CVD vs no CVD) |
| --- | --- | --- | --- | --- |
| No CVD | ACEi | 1659/272,630 (6·1) | *Reference* |  |
|  | ARB | 1040/196,202 (5·3) | 0·87 (0·80-0·94) | *Ref* |
|  | Beta blocker | 2128/373,265 (5·7) | 0·82 (0·76-0·89) | *Ref* |
|  | CCB | 1346/231,960 (5·8) | 0·80 (0·73-0·87) | *Ref* |
|  | Diuretic | 2463/435,515 (5·7) | 0·65 (0·64-0·82) | *Ref* |
| CVD | ACEi | 592/84,493 (7·0) | *Reference* |  |
|  | ARB | 306/49,805 (6·1) | 0·85 (0·74-0·97) | 0·98 |
|  | Beta blocker | 861/135,829 (6·3) | 0·72 (0·64-0·81) | 0·03 |
|  | CCB | 413/68,214 (6·0) | 0·71 (0·62-0·81) | 0·09 |
|  | Diuretic | 6704/98,204 (7·2) | 0·69 (0·61-0·78) | 0·27 |
| Subgroup | **AHM-class** | **Dementia cases/total person years^b^ n(‰)** | **HR^c^ (95%CI)** | **P-value interaction**  **(CHF vs no CHF)** |
| No CHF | ACEi | 1911/326,624 (5·9) | *Reference* |  |
|  | ARB | 1134/226,812 (5·0) | 0·83 (0·77-0·89) | *Ref* |
|  | Beta blocker | 2429/460,035 (5·3) | 0·77 (0·72-0·83) | *Ref* |
|  | CCB | 1544/279,477 (5·5) | 0·77 (0·71-0·84) | *Ref* |
|  | Diuretic | 2486/475,382 (5·2) | 0·66 (0·61-0·71) | *Ref* |
| CHF | ACEi | 340/30,499 (11·2) | *Reference* |  |
|  | ARB | 212/19,195 (11·0) | 1·02 (0·87-1·20) | 0·02 |
|  | Beta blocker | 560/49,058 (11·4) | 1·02 (0·87-1·19) | <0·001 |
|  | CCB | 215/20,697 (10·4) | 0·77 (0·65-0·92) | 0·93 |
|  | Diuretic | 681/58,337 (11·7) | 0·62 (0·52-0·75) | 0·57 |

^a^ Cardiovascular disease is a composite of history of heart attack and/or stroke ^b^ Within class of interest ^c^ Model 2, adjusted for baseline age, sex, number of AHM-classes simultaneously used, type 2 diabetes, myocardial infarction and stroke as time dependent variables. Individual participants may be represented in multiple AHM-classes in case of combination therapy and medication switching over time. Similarly, patients may switch subgroup after developing diabetes, CVD or congestive heart failure over time. AHM = antihypertensive medication; HR = hazard ratio; CI = confidence interval; P-int = p for interaction between the two subgroups; CVD = cardiovascular disease; CHF = congestive heart failure; ACEi = angiotensin-converting enzyme inhibitor; ARB = angiotensin receptor blocker; CCB = calcium channel blocker

**Supplementary Table 12. Subgroup analysis stratified for number of simultaneously used AHM-classes; association between AHM classes and dementia, compared to use of ACEi**

| **Number of AHM classes used simultaneously** | **AHM-class** | **Dementia cases/**  **total person years^a^ n(‰)** | **HR^b^ (95%CI)** | **P-value interaction^c^**  **(versus “One” AHM users)** |
| --- | --- | --- | --- | --- |
| One | ACEi | 477/79,466 (6·0) | *Ref* |  |
|  | ARB | 225/42,686 (5·3) | 0·83 (0·71–0·98) | *Ref* |
|  | Beta blocker | 622/136,309 (4·6) | 0·76 (0·68–0·86) | *Ref* |
|  | CCB | 304/51,327 (5·9) | 0·84 (0·73–0·97) | *Ref* |
|  | Diuretic | 615/98,218 (6·3) | 0·68 (0·60–0·77) | *Ref* |
| Two | ACEi | 853/130,345 (6·5) | *Ref* |  |
|  | ARB | 465/84,186 (5·5) | 0·85 (0·76–0·95) | 0·86 |
|  | Beta blocker | 1042/157,875 (6·6) | 0·84 (0·75–0·94) | 0·26 |
|  | CCB | 530/83,019 (6·4) | 0·75 (0·67–0·86) | 0·27 |
|  | Diuretic | 1160/198,724 (5·8) | 0·62 (0·55–0·70) | 0·98 |
| Three | ACEi | 686/105,454 (6·5) | *Ref* |  |
|  | ARB | 456/79,351 (5·8) | 0·88 (0·78–1·00) | 0·57 |
|  | Beta blocker | 962/146,701 (6·6) | 0·84 (0·69–1·03) | 0·41 |
|  | CCB | 576/99,030 (5·8) | 0·76 (0·63–0·91) | 0·37 |
|  | Diuretic | 1028/167,212 (6·2) | 0·68 (0·55–0·84) | 0·98 |
| Four | ACEi | 216/37,431 (5·8) | *Ref* |  |
|  | ARB | 181/35,356 (5·1) | 0·87 (0·69–1·08) | 0·99 |
|  | Beta blocker | 344/63,781 (5·4) | 0·74 (0·39–1·41) | 0·94 |
|  | CCB | 330/62,370 (5·3) | 0·61 (0·39–0·94) | 0·17 |
|  | Diuretic | 345/65,139 (5·3) | 0·37 (0·19–0·73) | 0·09 |

^a^ Within class of interest ^b^ Model 2 adjusted (for baseline age, sex, number of AHM-classes used simultaneously, type 2 diabetes, myocardial infarction and stroke) ^c^ Compared to simultaneous use of one AHM class.

AHM = antihypertensive medication; ACEi = angiotensin-converting enzyme inhibitor; HR = hazard ratio; CI = confidence interval; P-int = p for interaction compared to simultaneous use of one AHM class. ARB = angiotensin receptor blocker; CCB = calcium channel blocker

**Supplementary Table 13. Subgroup analysis stratified for baseline age-group; association between AHM-classes and dementia, compared to use of ACEi**

| Age category | AHM-class | Dementia cases/total person years^b^ n(‰) | HR (95%CI) | P-value interaction  (versus <60 years) | P-interaction-trend^c^ (age category as ordinal) |
| --- | --- | --- | --- | --- | --- |
| <60 years | ACEi | 273/211,430 (1.3) | *Reference* |  |  |
|  | ARB | 135/142,783 (1·0) | 0·71 (0·58-0·86) | *Ref* |  |
|  | Beta blocker | 292/296475 (1·0) | 0·67 (0·57-0·79) | *Ref* |  |
|  | CCB | 187/166,722 (1·1) | 0·82 (0·68-0·98) | *Ref* |  |
|  | Diuretic | 302/293,740 (1·0) | 0·71 (0·61-0·84) | *Ref* |  |
| 60 – 70 years | ACEi | 876/102,431 (8·6) | *Reference* |  |  |
|  | ARB | 512/73,131 (7·0) | 0·80 (0·72-0·88) | 0·28 | 0·02 |
|  | Beta blocker | 1083/144,618 (7·5) | 0·76 (0·69-0·84) | 0·15 | 0·01 |
|  | CCB | 687/92,077 (7·5) | 0·79 (0·71-0·88) | 0·75 | 0·12 |
|  | Diuretic | 1096/158,026 (6·9) | 0·66 (0·60-0·73) | 0·39 | 0·12 |
| > 70 years | ACEi | 1102/43,262 (25·5) | *Reference* |  |  |
|  | ARB | 699/30,092 (23·2) | 0·94 (0·86-1·03) | 0·07 |  |
|  | Beta blocker | 1614/68,000 (23·7) | 0·89 (0·82-0·98) | 0·01 |  |
|  | CCB | 885/41,375 (21·4) | 0·75 (0·68-0·82) | 0·33 |  |
|  | Diuretic | 1769/81,954 (21·6) | 0·67 (0·62-0·74) | 0·42 |  |

^a^ Within class of interest ^b^ Model 2, adjusted for baseline age, sex, number of AHM-classes used simultaneously, type 2 diabetes, myocardial infarction and stroke as time dependent variables. Individual participants may be represented in multiple AHM-classes in case of combination therapy and medication switching over time ^c^ P-trend for interaction term with the three categories as an ordinal variable from <60, to 60-70, to >70 years

AHM = antihypertensive medication; ACEi = angiotensin-converting enzyme inhibitor; HR = hazard ratio; P-int = p for interaction between individual strata; CI = confidence interval; ARB = angiotensin receptor blocker; CCB = calcium channel blocker

**Supplementary Table 14-I. Analysis for individuals with their first AHM prescription starting 2015 or later: association between AHM-classes and dementia, compared to use of ACEi**

| **AHM-class** | **Dementia cases/**  **total person years^a^ n(‰)** | **HR^b^ Dementia (95%CI)** | **P-value Dementia** | **Mortality cases/**  **total person years^b^ n(‰)** | **HR^b,c^ Mortality**  **(95%CI)** | | **P-value**  **Mortality** |
| --- | --- | --- | --- | --- | --- | --- | --- |
| ACEi | 273/30,758 (8·9) | *Reference* | | 549/28,547 (19·2) | *Reference* | | |
| ARB | 132/17,272 (7·6) | 0·78 (0·63-0·97) | 0·025 | 240/16,268 (14·8) | 0·73 (0·62-0·85) | 0·0001 | |
| Beta blocker | 352/35,996 (9·8) | 0·94 (0·76-1·15) | 0·34 | 863/32,363 (26·7) | 1·56 (1·36-1·76) | <0·001 | |
| CCB | 204/28,247 (7·2) | 0·71 (0·57-0·89) | 0·003 | 448/26,205 (17·1) | 1·07 (0·91-1·24) | 0·38 | |
| Diuretic | 347/36,917 (9·4) | 0·77 (0·62-0·95) | 0·02 | 1173/33,655 (34·9) | 2·75 (2·40-3·16) | <0·001 | |

^a^ Within class of interest ^b^ Model 2 adjusted (for baseline age, sex, number of AHM-classes used simultaneously, type 2 diabetes, myocardial infarction and stroke). Individual participants may be represented in multiple AHM-classes in case of combination therapy and medication switching over time.

AHM = antihypertensive medication; ACEi = angiotensin-converting enzyme inhibitor; HR = hazard ratio; P-int = p for interaction between individual strata; CI = confidence interval; ARB = angiotensin receptor blocker; CCB = calcium channel blocker

**Supplementary Table 14-II. Analysis for individuals with their first AHM prescription before 2015: association between AHM-classes and dementia, compared to use of ACEi**

| **AHM-class** | **Dementia cases/**  **total person years^a^ n(‰)** | **HR^b^ Dementia (95%CI)** | **P-value Dementia** | **Mortality cases/**  **total person years^b^ n(‰)** | **HR^b,c^ Mortality**  **(95%CI)** | | **P-value**  **Mortality** |
| --- | --- | --- | --- | --- | --- | --- | --- |
| ACEi | 1,978/326,365 (6·1) | *Reference* | | 4,417/299,599 (14·7) | *Reference* | | |
| ARB | 1,214/228,735 (5·3) | 0·87 (0·81-0·93) | 0·001 | 2,582/207,830 (12·4) | 0·84 (0·80-0·89) | <0·001 | |
| Beta blocker | 2,637/473,097 (5·6) | 0·79 (0·73-0·86) | <0·001 | 6,800/426,309 (16·0) | 1·21 (1·15-1·28) | <0·001 | |
| CCB | 1,555/271,927 (5·7) | 0·78 (0·71-0·85) | <0·002 | 3,781/251,147 (15·1) | 1·06 (1·00-1·12) | 0·06 | |
| Diuretic | 2,820/496,802 (5·7) | 0·64 (0·59-0·70) | <0·003 | 8,256/449,857 (18·4) | 1·67 (1·59-1·79) | <0·001 | |

^a^ Within class of interest ^b^ Model 2 adjusted (for baseline age, sex, number of AHM-classes used simultaneously, type 2 diabetes, myocardial infarction and stroke). Individual participants may be represented in multiple AHM-classes in case of combination therapy and medication switching over time.

AHM = antihypertensive medication; ACEi = angiotensin-converting enzyme inhibitor; HR = hazard ratio; P-int = p for interaction between individual strata; CI = confidence interval; ARB = angiotensin receptor blocker; CCB = calcium channel blocker

**Supplementary Table 15-I. Subgroup analyses stratified for sex, and diabetes; associations between use of AHM-classes and mortality, compared to use of ACEi**

| Subgroup | AHM-class | Mortality cases/total person years^a^ n(‰) | HR^b^ (95%CI) | P-value interaction  (men vs women) |
| --- | --- | --- | --- | --- |
| Women | ACEi | 2373/164,123 (14·5) | *Reference* |  |
|  | ARB | 1573/136,483 (11·5) | 0·85 (0·80-0·90) | *Ref* |
|  | Beta blocker | 3890/272,040 (14·3) | 1·17 (1·11-1·25) | *Ref* |
|  | CCB | 2220/155,933 (14·2) | 1·05 (0·99-1·12) | *Ref* |
|  | Diuretic | 5110/313,049 (16·3) | 1·60 (1·50-1·70) | *Ref* |
| Men | ACEi | 2767/184,173 (15·0) | *Reference* |  |
|  | ARB | 1373/104,771 (13·1) | 0·81 (0·76-0·87) | 0·37 |
|  | Beta blocker | 3962/222,297 (17·8) | 1·23 (1·16-1·31) | 0·17 |
|  | CCB | 2137/137,164 (15·6) | 1·06 (0·97-1·11) | 0·72 |
|  | Diuretic | 4416/206,238 (21·4) | 1·80 (1·69-1·92) | 0·001 |
| Subgroup | **AHM-class** | **Dementia cases/total person years^b^ n(‰)** | **HR^c^ (95%CI)** | **P-value interaction**  **(CHF vs no CHF)** |
| No CHF | ACEi | 1911/326,624 (5·9) | *Reference* |  |
|  | ARB | 1134/226,812 (5·0) | 0·83 (0·77-0·89) | *Ref* |
|  | Beta blocker | 2429/460,035 (5·3) | 0·77 (0·72-0·83) | *Ref* |
|  | CCB | 1544/279,477 (5·5) | 0·77 (0·71-0·84) | *Ref* |
|  | Diuretic | 2486/475,382 (5·2) | 0·66 (0·61-0·71) | *Ref* |
| CHF | ACEi | 340/30,499 (11·2) | *Reference* |  |
|  | ARB | 212/19,195 (11·0) | 1·02 (0·87-1·20) | 0·02 |
|  | Beta blocker | 560/49,058 (11·4) | 1·02 (0·87-1·19) | <0·001 |
|  | CCB | 215/20,697 (10·4) | 0·77 (0·65-0·92) | 0·93 |
|  | Diuretic | 681/58,337 (11·7) | 0·62 (0·52-0·75) | 0·57 |

^a^ Within class of interest ^b^ Model 2, adjusted for baseline age, sex, number of AHM-classes simultaneously used, type 2 diabetes, myocardial infarction and stroke as time dependent variables. Individual participants may be represented in multiple AHM-classes in case of combination therapy and medication switching over time. Similarly, patients may switch subgroup after developing diabetes, CVD or congestive heart failure over time. AHM = antihypertensive medication; HR = hazard ratio; CI = confidence interval; P-int = p for interaction between the two subgroups; CVD = cardiovascular disease; CHF = congestive heart failure; ACEi = angiotensin-converting enzyme inhibitor; ARB = angiotensin receptor blocker; CCB = calcium channel blocker

**Supplementary Table 15-II. Subgroup analyses stratified for cardiovascular disease** ^a^ **and congestive heart failure; association between use of AHM-classes and mortality, compared to use of ACEi**

| Subgroup | AHM-class | Mortality cases/total person years^b^ n(‰) | HR^c^ (95%CI) | P-value interaction  (CVD vs no CVD) |
| --- | --- | --- | --- | --- |
| No CVD | ACEi | 1650/82,828 (19·9) | *Reference* |  |
|  | ARB | 906/49,045 (18·5) | 0·86 (0·80-0·93) | *Ref* |
|  | Beta blocker | 2831/132,859 (19·3) | 1·19 (1·12-1·31) | *Ref* |
|  | CCB | 1294/67,056 (19·3) | 0·98 (0·92-1·07) | *Ref* |
|  | Diuretic | 2805/96,317 (29·1) | 1·99 (1·85-2·15) | *Ref* |
| CVD | ACEi | 3490/265,467 (13·2) | *Reference* |  |
|  | ARB | 2040/192,209 (10·6) | 0·81 (0·78-0·86) | 0·26 |
|  | Beta blocker | 5021/361,482(13·9) | 1·20 (1·14-1·27) | 0·77 |
|  | CCB | 3063/226,041 (13·6) | 1·07 (1·01-1·14) | 0·03 |
|  | Diuretic | 6721/422,970 (15·9) | 1·59 (1·50-1·68) | <0·001 |
| Subgroup | **AHM-class** | **Mortality cases/total person years^b^ n(‰)** | **HR^c^ (95%CI)** | **P-value interaction**  **(CHF vs no CHF)** |
| No CHF | ACEi | 3504/318,295 (11·0) | *Reference* |  |
|  | ARB | 2050/222,271 (9·2) | 0·82 (0·78-0·87) | *Ref* |
|  | Beta blocker | 5195/445,930 (11·7) | 1·15 (1·09-1·21) | *Ref* |
|  | CCB | 3374/272,718 (12·4) | 1·16 (1·09-1·23) | *Ref* |
|  | Diuretic | 5793/461,983 (12·5) | 1·41 (1·33-1·49) | *Ref* |
| CHF | ACEi | 1636/30,001 (54·5) | *Reference* |  |
|  | ARB | 896/18,982 (47·2) | 0·92 (0·85-1·00) | 0·01 |
|  | Beta blocker | 2657/48,411 (54·9) | 1·15 (1·06-1·24) | 0·91 |
|  | CCB | 983/20,380 (48·2) | 0·93 (0·85-1·01) | <0·001 |
|  | Diuretic | 3733/57,304 (65·1) | 1·96 (1·76-2·19) | <0·001 |

^a^ Cardiovascular disease is a composite of history of heart attack and/or stroke ^b^ Within class of interest ^c^ Model 2, adjusted for baseline age, sex, number of AHM-classes simultaneously used, type 2 diabetes, myocardial infarction and stroke as time dependent variables. Individual participants may be represented in multiple AHM-classes in case of combination therapy and medication switching over time. Similarly, patients may switch subgroup after developing diabetes, CVD or congestive heart failure over time. AHM = antihypertensive medication; HR = hazard ratio; CI = confidence interval; P-int = p for interaction between the two subgroups; CVD = cardiovascular disease; CHF = congestive heart failure; ACEi = angiotensin-converting enzyme inhibitor; ARB = angiotensin receptor blocker; CCB = calcium channel blocker

**Supplementary Table 16. Subgroup analysis stratified for number of simultaneously used AHM-classes; association between AHM classes and mortality, compared to use of ACEi**

| **Number of AHM classes used simultaneously** | **AHM-class** | **Mortality cases/**  **total person years^a^ n(‰)** | **HR^b^ (95%CI)** | **P-value interaction^c^**  **(versus “One” AHM users)** |
| --- | --- | --- | --- | --- |
| One | ACEi | 667/75,247 (8·9) | *Ref* |  |
|  | ARB | 307/40,788 (7·5) | 0·87 (0·76-0·99) | *Ref* |
|  | Beta blocker | 1158/126,674 (9·1) | 1·06 (0·97-1·17) | *Ref* |
|  | CCB | 574/47,842 (12,0) | 1·24 (1·11-1·38) | *Ref* |
|  | Diuretic | 1779/90,497 (19·7) | 1·80 (1·65-1·97) | *Ref* |
| Two | ACEi | 1609/127,464 (12·6) | *Ref* |  |
|  | ARB | 842/824,445 (10·2) | 0·85 (0·78-0·93) | 0·85 |
|  | Beta blocker | 2543 (154,880 (16·4) | 1·34 (1·25-1·45) | <0·001 |
|  | CCB | 1130/81,090 (13·9) | 1·21 (1·11-1·32) | 0·75 |
|  | Diuretic | 3332/194,477 (17·1) | 1·82 (1·68-1·97) | 0·90 |
| Three | ACEi | 2062/104,093 (19·8) | *Ref* |  |
|  | ARB | 1184/78,564 (15·1) | 0·82 (0·76-0·88) | 0·49 |
|  | Beta blocker | 2946/145,092 (20·3) | 1·22 (1·08-1·37) | 0·076 |
|  | CCB | 1509/97,880 (15·4) | 0·87 (0·79-0·97) | <0·001 |
|  | Diuretic | 3198/165278 (19·4) | 1·33 (1·16-1·51) | <0·001 |
| Four | ACEi | 745/37,107 (20·1) | *Ref* |  |
|  | ARB | 556/35,071 (15·9) | 0·77 (0·68-0·87) | 0·23 |
|  | Beta blocker | 1148/63,310 (18·3) | 0·85 (0·57-1·26) | 0·27 |
|  | CCB | 1087/61,899 (17·6) | 0·51 (0·40-0·64) | <0·001 |
|  | Diuretic | 1160/64,649 (17·9) | 0·70 (0·41-1·19) | <0·001 |

^a^ Within class of interest ^b^ Model 2 adjusted (for baseline age, sex, number of AHM-classes used simultaneously, type 2 diabetes, myocardial infarction and stroke) ^c^ Compared to simultaneous use of one AHM class.

AHM = antihypertensive medication; ACEi = angiotensin-converting enzyme inhibitor; HR = hazard ratio; CI = confidence interval; ARB = angiotensin receptor blocker; CCB = calcium channel blocker

**Supplementary Table 17. Subgroup analysis stratified for baseline age; association between AHM classes and mortality, compared to use of ACEi**

| Age category | AHM-class | Dementia cases/total person years^b^ n(‰) | HR (95%CI) | P-value interaction  (versus <60 years) | P-interaction-trend^c^ (age category as ordinal) |
| --- | --- | --- | --- | --- | --- |
| <60 years | ACEi | 942/207,077 (4·6) | *Reference* |  |  |
|  | ARB | 489/140,493 (3·5) | 0·74 (0·67-0·82) | *Ref* |  |
|  | Beta blocker | 1235/287,808 (4·3) | 1·02 (0·94-1·12) | *Ref* |  |
|  | CCB | 760/163,209 (4·7) | 1·11 (1·01-1·222) | *Ref* |  |
|  | Diuretic | 1386/287,170 (4·8) | 1·52 (1·39-1·66) | *Ref* |  |
| 60 – 70 years | ACEi | 1751/99,649 (17·6) | *Reference* |  |  |
|  | ARB | 1041/71,622 (14·5) | 0·81 (0·76-0·88) | 0·12 | 0·002 |
|  | Beta blocker | 2629/140,793 (18·7) | 1·24 (1·16-1·33) | <0·001 | <0·001 |
|  | CCB | 1496/89,835 (16·7) | 1·04 (0·97-1·12) | 0·23 | 0·10 |
|  | Diuretic | 2958/153,663 (19·3) | 1·65 (1·53-1·77) | 0·11 | <0·001 |
| > 70 years | ACEi | 2447/41,570 (58·9) | *Reference* |  |  |
|  | ARB | 1416/29,139 (48·6) | 0·88 (0·83-0·94) | 0·003 |  |
|  | Beta blocker | 3988/65,740 (60·7) | 1·24 (1·17-1·32) | <0·001 |  |
|  | CCB | 2101/40,053 (52·5) | 1·02 (0·96-1·09) | 0·09 |  |
|  | Diuretic | 5182/78,454 (66·1) | 1·81 1·70-1·94) | <0·001 |  |

^a^ Within the class of interest when used during final year of observation ^b^ Model 2 adjusted (for baseline age, sex, number of AHM-classes used simultaneously, type 2 diabetes, myocardial infarction and stroke). Individual participants may be represented in multiple AHM-classes in case of combination therapy and medication switching over time.

AHM = antihypertensive medication; ACEi = angiotensin-converting enzyme inhibitor; HR = hazard ratio; P-int = p for interaction between individual strata; P-trend = P for trend between the three strata; CI = confidence interval; ARB = angiotensin receptor blocker; CCB = calcium channel blocker.

**Supplementary Table 18.** Competing risk according sub-distribution hazards (Fine & Gray)

| **AHM-class** | **Cases/PY n (‰)** | **HR^a^ (95%CI)** | **P-value** |
| --- | --- | --- | --- |
| ACEi | 2251/463,154 (4·9) | *Reference* | |
| ARB | 1346/305,062 (4·4) | 0·90 (0·85-0·97) | 0·007 |
| Beta blocker | 2989/673,934 (4·4) | 0·80 (0·74-0·86) | <0·001 |
| CCB | 1759/389,583 (4·5) | 0·80 (0·74-0·87) | <0·001 |
| Diuretic | 3167/728,310 (4·4) | 0·60 (0·56-0·65) | <0·001 |

^a^ Model 2 , adjusting for age and sex at baseline, and number of AHM-classes simultaneously used, type 2 diabetes, myocardial infarction, and stroke as time-dependent variables. Abbreviations: AHM = antihypertensive medication; HR = hazard ratio; CI = confidence interval; ACEi = angiotensin-converting enzyme inhibitor; ARB = angiotensin receptor blocker, CCB = calcium channel blocker.
